# Unlocking the Zn-enriching potential of industrial yeast strains—an experimental journey from metal analysis to proteomics

**DOI:** 10.1007/s00253-025-13692-y

**Published:** 2026-01-10

**Authors:** Gina Grimmer, Julia Muenzner, Maximillian Schmacht, Maria Angels Subirana, Iris H. Valido, Philip Nickl, Paul M. Dietrich, Ievgen S. Donskyi, Dirk Schaumlöffel, Martin Hageböck, Michael Mülleder, Markus Ralser, Hajo Haase, Martin Senz, Maria Maares, Claudia Keil

**Affiliations:** 1 Department of Food Chemistry, Institute of Nutritional Science, University of Potsdam, Arthur-Scheunert-Allee 114–116, 14558 Nuthetal, Germany; 2 Department of Food Chemistry and Toxicology, Institute of Food Technology and Food Chemistry, Technische Universität Berlin, Straße des 17. Juni 135, 10623 Berlin, Germany; 3 Department Bioprocess Engineering and Applied Microbiology, Versuchs- und Lehranstalt für Brauerei in Berlin (VLB) e.V, Seestraße 13, 13353 Berlin, Germany; 4 Department of Biochemistry, Charité Universitätsmedizin Berlin, 10117 Berlin, Germany; 5 CNRS, Université de Pau et des Pays de l’Adour, Institut des Sciences Analytiques et de Physico-Chimie pour l’Environnement et les Matériaux (IPREM) UMR 5254, Université de Pau et des Pays de l’Adour, Hélioparc, 2 avenue Pierre Angot, 64053 Pau, France; 6 GTS Research Group, Department of Chemistry, Faculty of Science, Universitat Autònoma de Barcelona, Cerdanyola del Vallès, 08193 Barcelona, Spain; 7 Institut für Chemie und Biochemie, Freie Universität Berlin, Takustr. 3, 14195 Berlin, Germany; 8 SPECS Surface Nano Analysis GmbH, Voltastrasse 5, 13355 Berlin, Germany; 9 Core Facility – High Throughput Mass Spectrometry, Charité Universitätsmedizin Berlin, Berlin, Germany; 10 Centre for Human Genetics, Nuffield Department of Medicine, University of Oxford, Oxford, UK; 11 Berlin Institute of Health at Charité - Universitätsmedizin Berlin, Berlin, Germany; 12 Max Planck Institute for Molecular Genetics, Ihnestrasse 73, 14195 Berlin, Germany

**Keywords:** Zinc supplement, Zinc-enriched yeast, Proteomics, Zinc speciation, XAS

## Abstract

**Abstract:**

Nutritional supplements such as trace element-enriched yeasts are becoming increasingly popular to overcome the worldwide problem of zinc (Zn) deficiency. Unlike selenium-enriched yeast, which is already authorized in the European Union, Zn-enriched yeasts (ZnY) have not yet been approved for food purposes in the European Union, as their evaluation is still ongoing, demanding more comprehensive data regarding the Zn species present in ZnY. This study screens ten different industrial yeast strains regarding their Zn-enrichment quota, with further characterization of selected strains using spectroscopic and proteomic approaches. Microfermentation experiments on the industrial yeasts showed Zn levels spanning 0.06–51 pg/cell. Large-scale fermentation in bioreactors was carried out with two strains excelling in either biomass or Zn accumulation. A combination of inductively coupled plasma mass spectrometry (ICP-MS) and various spectroscopic methods confirmed the Zn enrichment, while suggesting that fractions of the Zn accumulated on the cell surface, with simultaneously high values of phosphorus being present. Speciation via X-ray absorption spectroscopy (XAS) analyses revealed that Zn species are transformed and Zn is coordinated to P-O-ligands and to amino acid ligands in both strains. Proteomic analysis showed that ZnY cells moved from a Zap1-governed Zn balance to an intracellular excess response, implying cellular Zn uptake. This study demonstrates that, in a Zn-excess medium, industrial yeast strains exhibit variability in Zn-accumulation capacity, cellular Zn-localization, and regulatory responses involving the expression of Zn-binding proteins. The presented findings contribute to optimizing industrial fermentation processes for producing Zn-rich yeast biomass and enhance the understanding of Zn regulation in yeast, aiding in the approval of Zn-enriched yeasts for supplements and novel food applications.

**Key points:**

• *Zn enrichment in yeasts is strongly time and strain dependent*

• *Zn proteome changes under Zn excess suggest that Zn is partly internalized in the yeast cells*

• *Beside proteins, phosphorous compounds seem to be Zn-binding ligands in Zn-enriched yeast*

**Graphical abstract:**

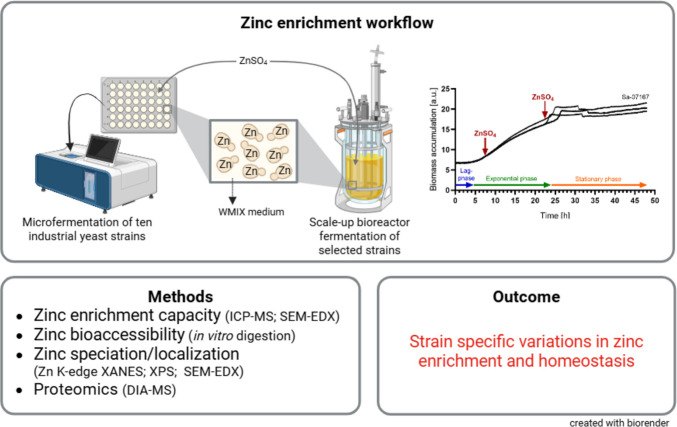

**Supplementary information:**

The online version contains supplementary material available at 10.1007/s00253-025-13692-y.

## Introduction

The use of yeast in food processing and fermenting alcoholic beverages is traditionally seen as the earliest innovation in biotechnology, going back thousands of years (Lahue et al. 2020). Currently, over 2000 yeast species in more than 100 genera are identified, with public collections housing thousands of strains (Boundy-Mills et al. 2016; Boekhout et al. 2022) for potential use in food, medicine, and agriculture (Niego et al. 2023; Geijer et al. 2022; Parapouli et al. 2020; Steensels et al. 2014). Besides the traditional application of yeasts in baking and brewing, yeast-based ingredients nowadays are also utilized in food manufacturing to enhance flavors, adjust aromas, and cover unwanted tastes in various products (Tomé 2021; Tao et al. 2023). Furthermore, yeasts and yeast autolysates are gaining attention as dietary supplements and functional foods due to their promising health advantages (Jach et al. 2015; EFSA 2011, 2021; Rai et al. 2019). The focus on mineral-enriched yeast for optimizing trace element intake has intensified in recent years. Selenium (Se)- and copper (Cu)-enriched yeasts are already among the globally available food supplements (Meena et al. 2020; Sun et al. 2022). Marketing such products is subject to inconsistent legal frameworks worldwide (Thakkar et al. 2020). In the European Union, their authorization is governed by Directive 2002/46/EC (European Parliament and the Council 2002), which requires an assessment of their safety by the European Food Safety Authority (EFSA). The EFSA so far only approved the use of Se-enriched yeast biomass for nutritional purposes (EFSA 2008, 2020). The evaluation of Zn-enriched yeasts (ZnY), first requested in 2009, is still ongoing as EFSA demands more comprehensive data on their composition and Zn-bioavailability after intestinal digestion and uptake (EFSA 2009a, 2009b, 2011). Cost-effective ZnY production is achieved by cultivating yeast in a Zn-enriched environment, where controlled Zn levels ensure incorporation into the biomass without reaching toxicity. Yeast can accumulate Zn through mechanisms of bioadsorption, biomineralization and/or intracellular bioaccumulation, each leaving a distinct Zn speciation signature. The first two processes involve passive physicochemical interactions, allowing Zn ions to bind either to anionic groups on the yeast surface or react with microbial exudates, leading to the formation of Zn deposits on the yeast surface (Chen and Wang 2008; Gadd 2021; Sun et al. 2022). In contrast, the bioaccumulation of Zn inside the cell is an active, metabolism-driven process. Zn uptake across the plasma membrane is facilitated by carriers including the high-affinity transporter Zrt1 and the lower-affinity transporters Zrt2, Fet4, and Pho84. To maintain Zn homeostasis, *Saccharomyces cerevisiae* detoxifies by shuttling excess Zn from the cytosol to the vacuole (the Zrc1 and Cot1 proteins have been identified as Zn transporters into the yeast vacuole) or into organelles of the secretory pathway (Msc2/Zrg17 complex mediates Zn transport into the endoplasmic reticulum (ER)) (Bird and Wilson 2020; Eide 2020). Within vacuoles, long-chain polyphosphates and organic acids are discussed as important Zn-binding ligands that contribute to its storage (Simm et al. 2007). Furthermore, yeast controls Zn homeostasis by changing the abundance of Zn-binding proteins and peptides like glutathione and phytochelatins that contribute to cellular Zn buffering (Wang et al. 2018; Sun et al. 2022; Aulakh et al. 2025). These homeostatic and adaptive response mechanisms enable yeast to compensate Zn surplus while ensuring a minimal cellular Zn quota in low-Zn environments (MacDiarmid et al. 2000; Wang et al. 2018). The transcription factor Zap1 plays a pivotal role in this homeostatic network by regulating the expression of essential genes associated with Zn metabolism (Lyons et al. 2000; Wu et al. 2008; Eide 2009; Wang et al. 2018). Notably, most of the research that underpins our understanding of Zn homeostasis in yeast has been conducted using *Saccharomyces cerevisiae* and *Schizosaccharomyces pombe* reference strains or mutant libraries in controlled laboratory cultivation settings (Eide 2020; Zhao et al. 2020; Yao et al. 2023; Aulakh et al. 2025). There is a notable lack of studies examining how industrial yeast strains interact with and handle Zn and how they respond to metal ion perturbations. A few studies have addressed the impact of Zn in industrial fermentation settings (Nicola and Walker 2009; Nicola et al. 2009), chemostat cultures (Nicola et al. 2007), or in food-related non-*Saccharomyces* yeasts tested in the laboratory (Maares et al. 2022; Rossi et al. 2023).

This study aims to expand our knowledge on ZnY by looking at how industrial *Saccharomyces* strains accumulate Zn during micro- and batch bioreactor conditions. The yeast biomass was examined with various metal analysis methods, including fluorescence imaging, energy-dispersive X-ray spectroscopy (SEM-EDX), X-ray photoelectron spectroscopy (XPS), and X-ray absorption spectroscopy (XAS) to better understand Zn distribution and speciation in the samples. Proteome studies uncovered Zn-responsive proteins, giving valuable information about Zn regulation in those yeasts. To confirm ZnY suitability for food use, we applied an in vitro digestion model to test Zn solubility from the Zn-enriched yeast biomass. The presented findings contribute to optimizing industrial fermentation processes for producing Zn-rich yeast biomass and enhance the understanding of Zn regulation in yeast, aiding in the approval of yeast for supplements and novel food applications.

## Materials and methods

### Yeasts strains and medium

All yeast strains used in this work were taken from the strain collection of the Research and Teaching Institute for Brewing (VLB) in Berlin and chosen based on their industrial relevance and application (Table 1). The yeasts were first propagated from cryostocks onto YPD agar plates. The subsequent pre-cultures and main fermentation cultures were grown in a chemically defined WMIX medium (white molasses, version number IX) according to (Schmacht et al. 2017). Based on inductively coupled plasma mass spectrometry (ICP–MS) data, we estimated a basal level of 0.8 µmol/L Zn in the WMIX media.

**Table 1:** Overview of the yeast strains used

Genus	Species	Strain	Application	Origin and references
*Saccharomyces*	*cerevisiae* var. *boulardii*	Sa-0793	Probiotic yeast strain	VLB yeast bank
*Saccharomyces*	*cerevisiae* var. *boulardii*	Sa-07145	Probiotic yeast strain	VLB yeast bank
*Saccharomyces*	*pastorianus*	Rh	Bottom-fermenting brewer's yeast	VLB yeast bank (Maares et al. 2022)
*Saccharomyces*	*pastorianus*	Nr. 42	Bottom-fermenting brewer's yeast	VLB yeast bank
*Saccharomyces*	*cerevisiae*	Sa-0725	Top-fermenting brewer's yeast	VLB yeast bank
*Saccharomyces*	*cerevisiae*	Sa-0751	Distiller's yeast	VLB yeast bank
*Saccharomyces*	*cerevisiae*	Sa-0791	Sourdough yeast	VLB yeast bank
*Saccharomyces*	*cerevisiae*	Sa-07140	Top-fermenting brewer's yeast	VLB yeast bank; Type strain CBS1171 (Martini and Kurtzman 1985)
*Saccharomyces*	*cerevisiae*	Sa-07167	Baker's yeast	VLB yeast bank
*Saccharomyces*	*cerevisiae*	Sa-07346	glutathione production	VLB yeast bank (Lorenz et al. 2015)

### Fermentation conditions for Zn enrichment

The experimental runs in the screening process were conducted in a BioLector® Pro microfermentor (m2plabs GmbH, Baesweiler, Germany) (Funke et al. 2010; Blesken et al. 2016). The disposable 48-well flower plate microtiter format of the BioLector® Pro offers the possibility of online measurements of biomass by 620 nm scattered light measurement over the entire course of fermentation. The wells were inoculated from overnight WMIX pre-cultures with 1 × 10^7^ yeast cells/mL WMIX medium with a final fermentation volume of 1 mL and incubated under shaking (1200 rpm) at 26 °C. 10 mmol/L ZnSO_4_ was added either in the early or late exponential growth phase of the different strains. For each strain, a control sample was prepared with the addition of water instead of ZnSO_4_ in either the early or late exponential growth phase. Samples of cell culture media and cells were collected during fermentation (see Supplementary Information Table S1) to determine cell count, total Zn, and glucose concentration. Upscaled batch fermentations of *S. cerevisiae* Sa-07167 and *S. pastorianus* Nr. 42 were carried out in 5 L bioreactors (Biostat®A_plus_/Biostat®B, Sartorius AG, Göttingen, Germany) (Lorenz et al. 2015). The yeast cells were inoculated into a 1 L working volume of WMIX media in the bioreactor from overnight shake flask WMIX pre-cultures with 1 × 10^7^ yeast cells/mL. Cell samples were taken immediately to determine cell count, yeast dry mass, cell viability, and total Zn at the beginning. Fermentations were carried out at 26 °C with 200 rpm and 30% oxygen saturation, which was controlled by the stirring speed. Since the bioreactors were not equipped with an online cell monitoring system, samples were taken at intervals throughout fermentation to map the increase in biomass and to assess the viability of the cells. Screening with *S. cerevisiae* Sa-07167 revealed the highest Zn content when Zn was added during the early exponential growth phase. Thus, 10 mmol/L ZnSO_4_ was added 7.4 h after inoculating this strain into the bioreactor, and the cells were harvested one hour later. For strain *S. pastorianus* Nr. 42, Zn was added 6.7 h after fermentation start, and cells were harvested after 48 h of fermentation, as the highest Zn contents per biomass were achieved under these conditions during screening. Yeast suspensions were centrifuged after fermentation, pellets were washed with 50 mmol/L HEPES buffer, and freeze-dried (Sublimator 15, Zirbus technology GmbH, Bad Grund (Harz), Germany).

### Biomass and cell viability analysis

Total cell concentrations and cell volumes were determined with a Beckman Multisizer™ 3 Coulter Counter® (Beckman Coulter GmbH, Krefeld, Germany) with a capillary diameter of 30 µm. Cell viability was examined via flow cytometry (CyFlow® Cube 8, Sysmex Deutschland GmbH, Norderstedt, Germany) following a live-dead staining with the Yeast Control™—Viability kit (Sysmex Partec GmbH, Görlitz, Germany) (Köhler et al. 2020). The simultaneous use of fluorescein diacetate (FDA) and propidium iodide (PI) allows the two-color identification of viable cells (fluorescein +/PI-), vital cells with impaired cell membrane integrity (fluorescein +/PI +), and dead cells (fluorescein-/PI +) (Kwolek-Mirek and Zadrag-Tecza 2014). Yeast dry weight was determined from 5 mL cell suspension pellets washed with 0.9% NaCl solution and dried at 100 °C (T6200, Heraeus Holding GmbH, Hanau, Germany) up to weight constancy. Medium glucose levels were determined via high performance liquid chromatography (HPLC) measurements (van Wyk et al. 2023).

### Zinc analysis in cell biomass

Total Zn from fresh BioLector® Pro and Biostat® fermentation samples as well as freeze-dried biomass from Biostat® bioreactor fermentations were measured using an Elan DRC II inductively coupled plasma-mass spectrometer (PerkinElmer Inc., Rodgau, Germany) as reported previously (Rossi et al. 2023; Maares et al. 2022). For quality control, we analyzed the reference material Fortified Breakfast Cereal (NIST-3233) (Sigma-Aldrich, Taufkirchen, Germany), obtaining 677 ± 56 mg Zn/kg compared to its certified value of 628 ± 16 mg Zn/kg.

The fluorescent probe Zinpyr-1 (50 µmol/L solution in loading buffer, 10 mmol/L HEPES, pH 7.35, 120 mmol/L NaCl, 5.4 mmol/L KCl, 5 mmol/L glucose, 1.3 mmol/L, CaCl_2_, 1 mmol/L MgCl_2_, 1 mmol/L NaH_2_PO_4_), which specifically binds Zn ions at nanomolar concentrations (Burdette et al. 2001), was used for live-cell Zn imaging with Hoechst 33,258 co-staining on an Axio Imager M1 microscope (Carl Zeiss Microscopy GmbH, Jena, Germany) (filter settings Zinpyr-1: λex 450–490 nm/λem 515–560 nm; Hoechst: λex 365 nm/λem 445/50 nm).

SEM–EDX of the freeze-dried biomass was taken with a JXA-8530 F Field Emission Electron Probe Microanalyzer (JEOL (Germany) GmbH, Freising, Germany) operating at 10 kV accelerate voltage with an average beam current of 420 pA under high vacuum mode (Maares et al. 2022).

UHV and NAP-XPS experiments were performed with an EnviroESCA spectrometer (SPECS Surface Nano Analysis GmbH, Berlin, Germany), equipped with a monochromatic Al Kα X-ray source (excitation energy of 1486.71 eV) and a PHOIBOS 150 hemispherical electron analyzer (Dietrich et al. 2019; 2025). Freeze-dried yeast samples for XPS analysis were prepared on indium foil. The spectra were measured in normal emission, and a source-to-sample angle of 60° was used. All spectra were acquired in fixed analyzer transmission (FAT) mode. The binding energy scale of the instrument was calibrated according to ISO 15472 (DIN ISO 15472:2020–05). For quantification, the survey spectra were acquired at ultra-high vacuum conditions with a pass energy of 100 eV, and the spectra were quantified utilizing the empirical sensitivity factors that were provided by SPECS Surface Nano Analysis GmbH (the sensitivity factors were corrected with the transmission function of the spectrometer). For charge compensation, the highly-resolved XP spectra were acquired under near-ambient pressure conditions at 5 mbar in a H_2_O vapor atmosphere with a pass energy of 50 eV, and the respective data were fitted using UNIFIT 2020 data processing software (Unifit Scientific Software GmbH, Leipzig, Germany). For fitting, a Shirley background and a Gaussian/Lorentzian sum function [peak shape model GL (30); 30% Gaussian/70% Lorentzian character] were used. If not denoted otherwise, the L-G mixing component was set to 0.30 for all carbon peaks and 0.40 for all heteroatom peaks. All binding energies were calibrated to the signal observed for the aliphatic C–C bond component (Ebind = 285 eV) if not stated otherwise.

XAS measurements of freeze-dried yeast samples were carried out at SAMBA beamline from SOLEIL Synchrotron (Gif-sur-Yvette, France). The monochromator was calibrated with a standard of Zn (K-edge 9659 eV) (Thompson et al. 2001). The measurements were performed in transmission and fluorescence modes using a liquid nitrogen cryostat at 77 K to avoid radiation damage. The beam size was set to 1 × 2 mm^2^ (vertical × horizontal). Samples and zinc references were pressed into 5 mm pellets using a hydraulic press. Zinc references were prepared from either commercially available Zn compounds (zinc oxide, zinc sulfate heptahydrate, zinc sulphide, zinc chloride, zinc phosphate, zinc (II) protoporphyrin IX, 5,10,15,20-tetraphenyl-21H,23H-porphine zinc, zinc nitrate hexahydrate, zinc acetate dihydrate, zinc citrate dihydrate, zinc oxalate, zinc malate). Or they were synthesized (zinc histidine, zinc methionine, zinc cysteine) by mixing a water solution of the amino acids and zinc nitrate (pH 4.0) at a molar ratio of 10:1 overnight at 4 °C followed by freeze drying. The zinc–bovine serum albumin reference was generated with a 1:1 ratio of zinc: bovine serum albumin. For each sample, 2 replicates were measured, performing over 100 scans per replicate, and the results were averaged. For data normalization and analysis by linear combination fitting, Fastosh and Athena software were used (Landrot 2018; Ravel and Newville 2005).

### Proteomics

#### Cultivation and sample preparation

Yeasts were grown in BioLector® Pro microfermentators in either control WMIX medium or WMIX spiked with 10 mmol/L ZnSO_4_ in the early exponential growth phase. *S. cerevisiae* Sa-07167 cells were collected after 1 h Zn incubation and *S. pastorianus* Nr. 42 were incubated for another 41.3 h. Cells were collected and washed with ice-cold 50 mmol/L HEPES (pH 5.2). Pellets were frozen with liquid nitrogen, and all samples were kept at −80 °C until protein extraction. Lysis buffer (7 M urea, 100 mmol/L ammonium bicarbonate, 100 µL per sample) was added to the frozen yeast pellets, a volume of glass beads equal to the size of the yeast pellet was added to the tubes, and samples were lysed by two cycles of beadbeating (5 min, 1,500 rpm, followed by 5 min on ice) using a homogenizer (Geno/Grinder® Spex, Fisher Scientific GmbH, Schwerte, Germany). 100 µL of supernatant were taken off the samples for further processing. Samples were reduced and alkylated by the addition of 10 µL of 55 mmol/L dithiothreitol (DTT) and an incubation for 60 min at 30 °C. Then, 10 µL of 120 mmol/L iodoacetamide were added to the samples incubated for 30 min at 22 °C in the dark. Subsequently, 380 µL of 100 mmol/L ammonium bicarbonate buffer were added to each sample, and samples were digested using 2 μg trypsin/LysC protease mix for 17 h at 37 °C. The digestion was stopped by the addition of 25 μL of 20% formic acid to each sample, and peptides were purified by solid-phase extraction as described previously (Messner et al. 2021) and eluted in 330 µL 50% acetonitrile. Peptides were completely dried using a vacuum concentrator and re-dissolved in 30 µL 0.1% formic acid. Peptides were stored at −80 °C.

#### Liquid chromatography − mass spectrometry analysis (LC − MS)

Tryptic peptides were analyzed by LC-tandem mass spectrometry (MS/MS) using a Q Exactive Plus mass spectrometer (Thermo Fisher Scientific, Bremen, Germany) and an Ultimate 3000 RSLCnano (Thermo Fisher Scientific) instrument. Digested yeast samples were trapped onto a guard column (PepMap C18, 5 mm × 300 μm × 5 μm, 100Ǻ, Thermo Fisher Scientific) and eluted from the analytical nano LC column (75 µm i.d. × 500 mm nano Acclaim PepMap C18, 2 μm; 100 Å; Thermo Fisher Scientific). The separation was done by a mobile phase from 0.1% formic acid (FA, Buffer A) and 80% acetonitrile with 0.1% FA (Buffer B) and applying a linear gradient from 115 min including an increase of buffer B from 3–45% in 90 min at a flow rate of 250 nL/min.

The MS instrument was operated in the data independent mode as follows: For the yeast samples, the Orbitrap was configured to acquire 25 × 24 m/z (covering 400–1000 m/z), precursor isolation window DIA spectra (17,500 resolution, AGC target 1e6, maximum inject time 60 ms, normalized HCD collision energy 27%) using an overlapping window pattern. Precursor MS spectra (m/z 400–1000) were analyzed with 35,000 resolutions after 60 ms accumulation of ions to a 1e6 target value in centroid mode. Additionally, the background ions m/z 445.1200 acted as a lock mass.

#### Pre-processing and analysis of proteomics data

Raw data were processed separately for each species using data-independent acquisition by neural networks (DIA-NN version 1.8.1; https://github.com/vdemichev/DiaNN) (Demichev et al. 2020) with the scan window radius set to 6 and MS2 and MS1 mass accuracies set to 20 and 10 ppm, respectively. A spectral library-free approach was used for both yeast *S. pastorianus* Nr. 42 and *S. cerevisiae* Sa-07167 samples, with reference proteomes downloaded from UniProt (The UniProt Consortium 2025) for peptide matching and protein annotation (UP00000231 for *S. cerevisiae*, UP000050240 for *Saccharomyces eubayanus*). *S. cerevisiae* Sa-07167 was run against the *S. cerevisiae* reference genome, yeast *S. pastorianus* Nr. 42 against a mixed species library consisting of the concatenated reference proteomes for *S. cerevisiae* and *S. eubayanus*, with species-specific protein inference enabled. The following settings were used in DIA-NN: precursor m/z 300–1800, precursor charge 1–4, peptide length 7–30, N-terminal methionine excision enabled, cysteine carbamidomethylation enabled, maximum number of missed cleavages of 1, in-silico protease digest at the amino acids lysine (K) and arginine (R).

Data for each strain were pre-processed separately in R. First, only proteotypic peptides were retained, and precursors with *q*.values ≥ 0.01 and global *q*.value ≥ 0.01 were removed. Next, only precursors detected in at least two out of three replicates were retained, and the dataset was filtered for precursors that are found in both the control samples (CTR, no ZnSO_4_ added during fermentation) and Zn condition. The resulting set of precursors (16,405 for *S. cerevisiae* Sa-07167, 10,572 for *S. pastorianus* Nr. 42) was used to quantify the proteins shared between the conditions (2871 for *S. cerevisiae* Sa-07167, 3540 for *S. pastorianus* Nr. 42) using the maxlfq() function as implemented in the DIA-NN R package (https://github.com/vdemichev/diann-rpackage).

*S. eubayanus* genes were mapped to *S. cerevisiae* orthologs based on the annotation provided in the Supplementary Information S1 File Table A in the paper published by La Cerda Garcia-Caro and colleagues (La Cerda Garcia-Caro et al. 2022). For *S. eubayanus* genes without a mapped ortholog, the systematic gene identifier was used throughout all analyses. Two genes, *HEK2* and *SMC2*, were mapped to multiple systematic *S. eubayanus* identifiers (*HEK2*: DI49_0147, DI49_1119; *SMC2*: DI49_1646, DI49_1689). Therefore, the systematic identifiers were used throughout all analyses for these proteins.

Proteins detected in solely the CTR or Zn condition (78 proteins and 302 proteins for *S. cerevisiae* Sa-07167, respectively, and 170 proteins and 1274 proteins for *S. pastorianus* Nr. 42, respectively) were identified based on precursors detected at least two out of three replicates. There were some proteins that were identified by different sets of precursors per condition per protein. Since these precursors did not pass the “shared between conditions” filter, they were not quantified and therefore omitted from the analysis (22 proteins for *S. cerevisiae* Sa-07167, 93 proteins for *S. pastorianus* Nr. 42).

All proteomic analyses were performed in R. Over-representation analyses for proteins detected in only the CTR or Zn condition, as well as gene-set enrichment analyses, were performed using WebGestaltR version 0.4.6 (Elizarraras et al. 2024). For yeast *S. pastorianus* Nr. 42, *S. cerevisiae* orthologs of *S. eubayanus* proteins were used in over-representation or gene set enrichment analysis (GSEA); *S. eubayanus* proteins without *S. cerevisiae* orthologs were omitted from the analyses (see protein mapping in the Supplementary Information S1 File Table A in (La Cerda Garcia-Caro et al. 2022)). Heatmaps were generated using ComplexHeatmap version 2.22.0 (Gu et al. 2016). ZAP1 targets were downloaded and annotated using the Saccharomyces Genome Database (SGD) (Engel et al. 2025). Differential expression analysis was performed using limma version 3.63.2 (Ritchie et al. 2015), with *p*-values adjusted using the Benjamini–Hochberg method.

### In vitro assessment of the Zn bioaccessibility

Zn bioaccessibility was determined with a static in vitro digestion method (Maares et al. 2022), a suitable methodology widely employed in many fields of food and nutritional sciences (Zhou et al. 2023). A total of 500 mg of freeze-dried yeast samples were incubated successively in simulated digestive fluids resembling the environment in the human mouth, stomach, and intestine. Detailed information on the composition of the digestion solutions is provided in Table 1 of (Böhmert et al. 2014). During digestion, amylase, pepsin, trypsin, and lipase activities were verified using amylopectin azure, albumin/bromophenol blue, azocasein, and *p*-nitrophenyl acetate, respectively, with photometric detection of the resulting cleavage products (Caro et al. 1986; Böhmert et al. 2014). The Zn content of microwave-digested supernatants (digest agent 1:1 mixture of ultrapure HNO_3_ (65%) and H_2_O_2_ (30%); Mars 6, CEM GmbH, Kamp-Lintfort, Germany) was subsequently quantified using ICP-MS.

### Statistical analysis

Statistical significance of experimental results was calculated by GraphPad Prism software version 8.02 (GraphPad Software Inc., San Diego, CA, USA) using the tests indicated in the respective figure legends.

## Results

### Screening industrial Saccharomyces strains for their Zn accumulation potential

**Fig. 1:**
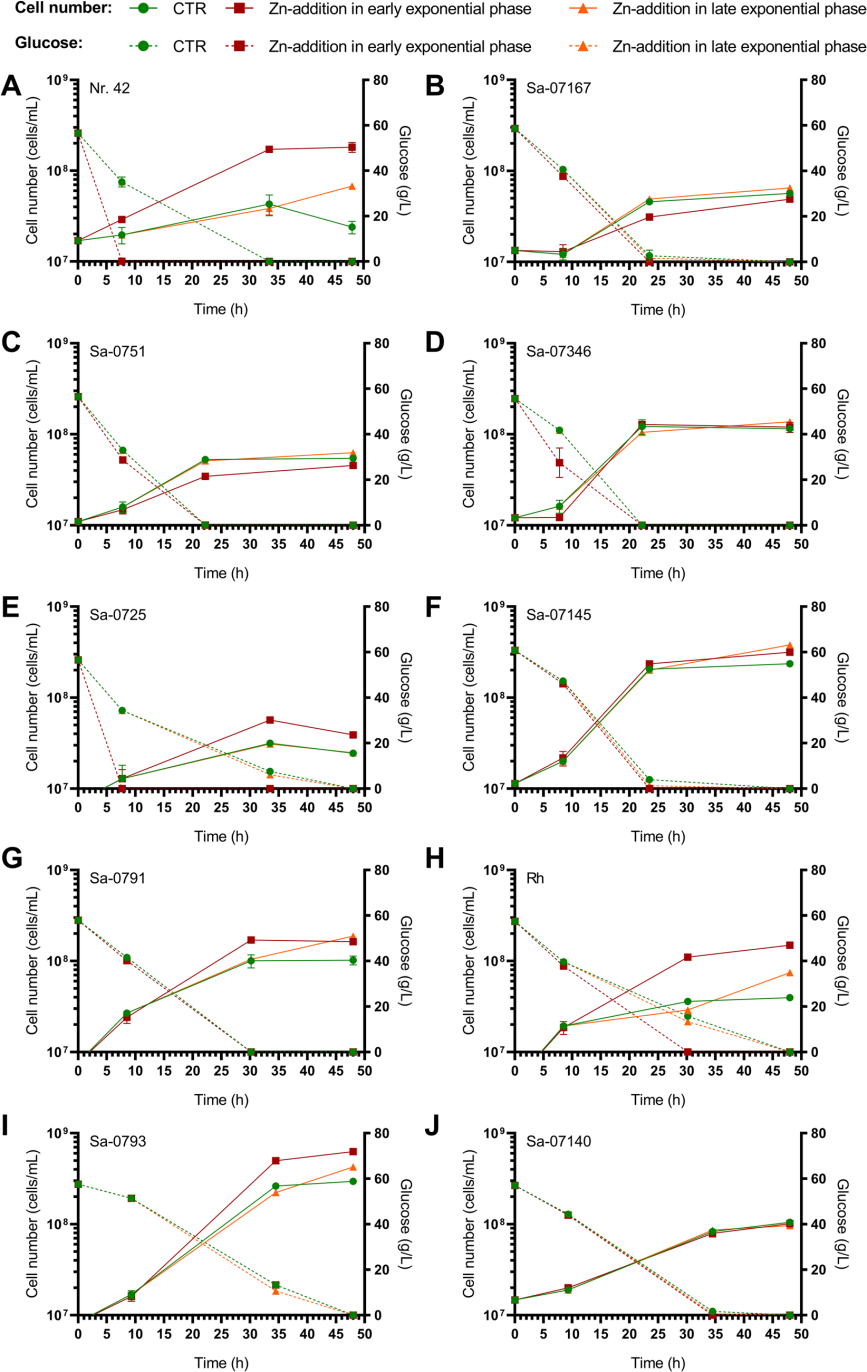
Growth kinetics and glucose consumption of yeasts during fermentation in the BioLector®Pro microbioreactor. Yeast strains were incubated in WMIX medium (CTR) or WMIX medium supplemented with 10 mmol/L ZnSO_4_ (ZnY) at the early exponential or late exponential growth phase, respectively. Details of the Zn incubation procedure during microfermentation are listed in Supplementary Information Table S1. The mean values ± SD of the cell concentrations and glucose consumption of CTR and ZnY during fermentation are shown

Industrial *Saccharomyces* strains (Table 1) were screened under microbioreactor conditions in WMIX growth medium for their ability to produce Zn-enriched biomass. Based on the strains’ growth curves, 10 mmol/L Zn sulphate (ZnSO_4_) was added at defined time points in the early or late exponential growth phase (Supplementary Information Fig. S1 and Table S1). A microscopic examination revealed some cell clustering in the Zn-treated yeast suspensions, while control samples (CTR) contained mostly separated cells (data not shown). These observations suggest Zn-binding to cationic cell surface molecules (β-glucan, chitin, and a mannoprotein-containing fibrillar outer layer), causing cell aggregation (flocculation). ZnY and CTR cultures reached similar final densities for most strains (Fig. 1). *S. pastorianus* strains Rh and Nr. 42 exhibited accelerated growth and increased glucose consumption when treated with Zn in the early exponential phase (Fig. 1). Live-dead fluorescence staining confirmed the yeast’s viability under CTR and Zn-excess fermentation conditions (~ 85–95% viability in CTR and ZnY).

**Fig. 2:**
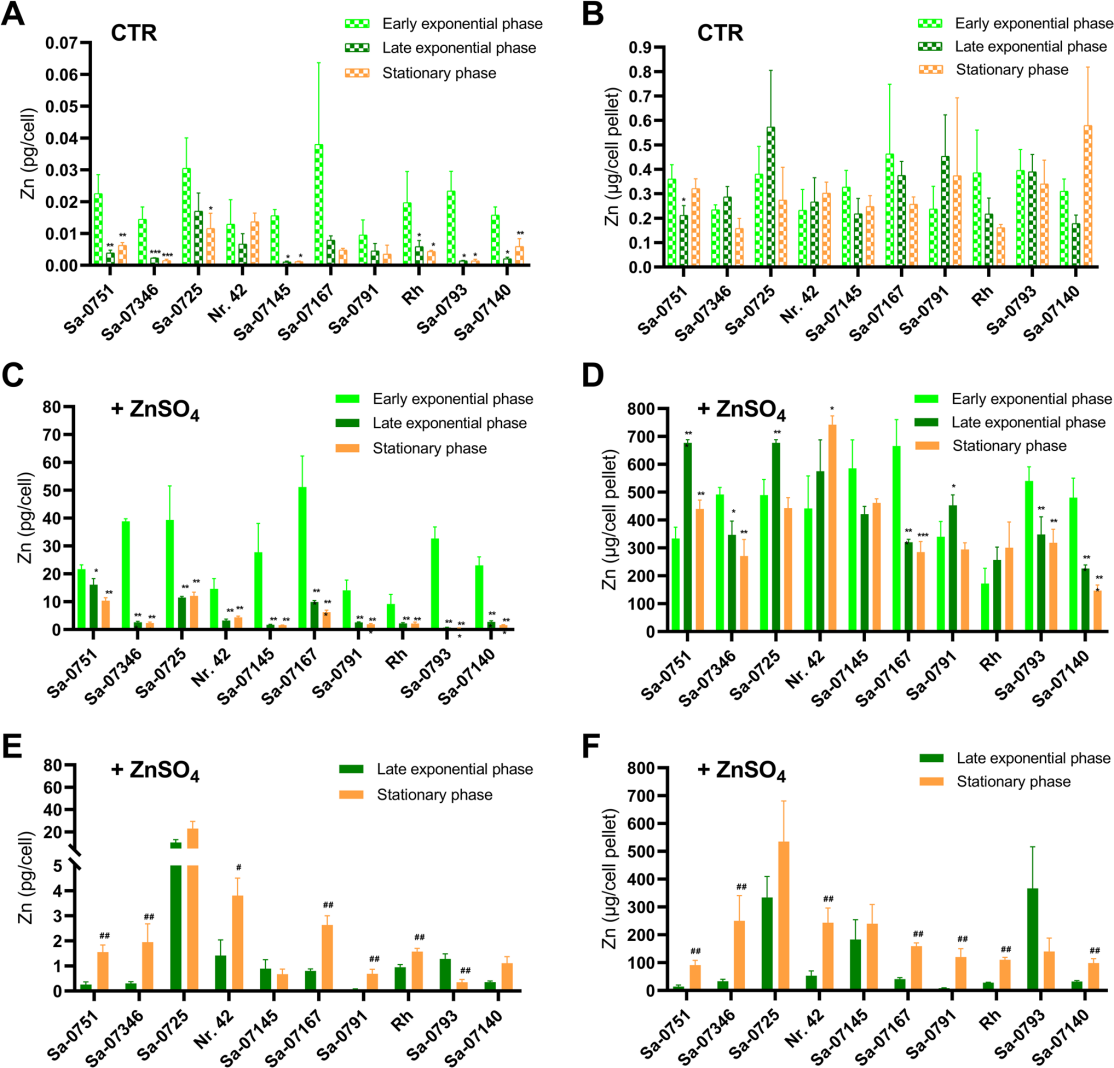
Zn enrichment of the yeasts during microfermentation in the BioLector®Pro microbioreactor. Yeast strains were fermented either in WMIX basal medium (CTR) (**A**/**B**) or WMIX medium supplemented with 10 mmol/L ZnSO_4_ in the early exponential (**C**/**D**) or late exponential (**E**/**F**) growth phase. The cells were harvested at the fermentation stages noted in the legend, which are shown in the figure as bars. Details of the Zn incubation procedure during microfermentation are listed in Supplementary Information Table S1. The Zn content of the biomass was determined by ICP-MS. Data (means ± SD of 3 replicates) are given as Zn content per cell (A/C/E). Total Zn per cell pellet (B/D/F) was estimated based on Zn-per-cell data and total cell counts from the 1 mL microfermentations (see Fig. 1 for details on cell concentrations). Statistics: **A**–**D** Significant differences from early exponential phase samples *p < 0.05, **p < 0.01, ***p < 0.001; two-way analysis of variance (ANOVA) with Dunnett’s multiple comparison test (E/F). Significant differences from early exponential phase samples Mann-Whitney *U* tests for statistical comparison # *p* < 0.05, ## *p* < 0.01

Zn content of the CTR yeasts was approximately in the range of 1–60 fg/cell (equivalent to ~ 1–55 × 10^7^ atoms of Zn per cell), which corresponds to a total quantity of approximately 0.2–0.8 µg Zn per yeast cell pellet obtained from 1 mL cell suspension in the microfermentations (Fig. 2A and B). A significant increase in per-cell Zn levels was observed in all ZnSO₄-treated yeasts. When Zn was added in the early exponential phase, cells displayed a peak in per-cell Zn levels after one hour, which then decreased over time (Fig. 2C). When treated with ZnSO_4_ during the late exponential phase, cells exhibited the highest Zn counts in stationary phase samples. Compared to early exponential Zn exposure, these yeast samples were notably lower in cellular Zn levels. The Zn content of ZnY samples varied slightly between strains but was typically in the range of pg/cell. The highest Zn counts per cell were found in *S. cerevisiae* Sa-07167 treated with ZnSO_4_ for 1 h in the early exponential phase (51.2 ± 11.2 pg Zn/cell; 665.8 ± 94.3 μg Zn/cell pellet; Fig. 2C and D). Due to the relatively high proliferation rates of *S. pastorianus* Nr. 42 during fermentation, this strain generated the highest Zn value per total cell mass (4.4 ± 0.4 pg Zn/cell; 742.0 ± 31.8 μg Zn/cell pellet; Fig. 2E and F).

### Scale-up of Zn-enriched yeast biomass production by batch bioreactor fermentations

**Fig. 3:**
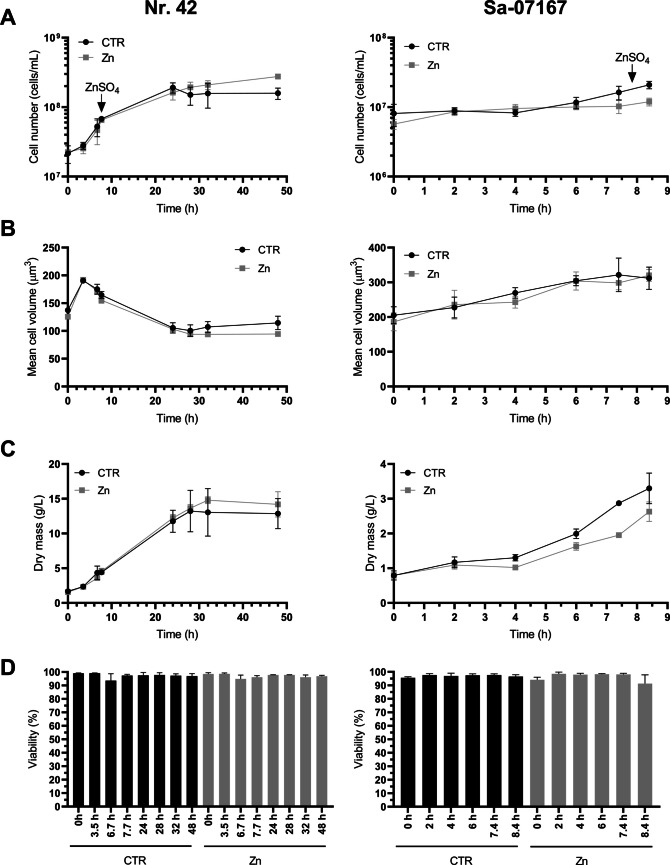
Overview of cell growth and viability of *S. pastorianus* Nr. 42 and *S. cerevisiae* Sa-07167 in an upscaled batch fermentation process. ZnSO_4_ was added to the yeast suspensions in the early exponential phase (*S. cerevisiae* Sa-07167 7.4 h and *S. pastorianus* Nr. 42 6.7 h after fermentation start). Cell concentration (**A**), average cell volume (**B**), and dry mass (**C**) were determined during fermentation. Yeast suspensions taken at the end of fermentations were tested for viability using a fluorescence-based live–dead assay. The results shown in (**D**) correspond to the fraction of fluorescein positive and propidium negative (FI^+^/PI^−^) cells. Data are shown as means ± SD of 3 replicates

Based on screening outcomes, *S. cerevisiae* Sa-07167 and *S. pastorianus* Nr. 42 were selected for scaled-up Zn enrichment using the Zn protocols that proved most effective in microfermentation tests, our approach was guided by industrial relevance, with each strain evaluated under conditions most likely encountered in practical fermentations at its peak Zn accumulation. Figure 3A-C illustrates the trends in cell number, cell volume, yeast dry mass, and cell viability during fermentation in the Biostat® bioreactors for both strains. As within the BioLector®Pro microbioreactor experiments, *S. cerevisiae* Sa-07167 suspensions only slightly increased in cell number over the total 8.4 h fermentation period in the Biostat® bioreactors. The average cell volumes and thus dry masses, however, increased in both CTR and ZnY. The biomass dynamics of CTR and ZnSO_4_-treated *S. pastorianus* Nr. 42 samples in the upscaled fermentation were almost comparable throughout the batch fermentation. Yeast samples collected from CTR and ZnY bioreactor fermentations were high in viability (> 90%) (Fig. 3D).

**Fig. 4:**
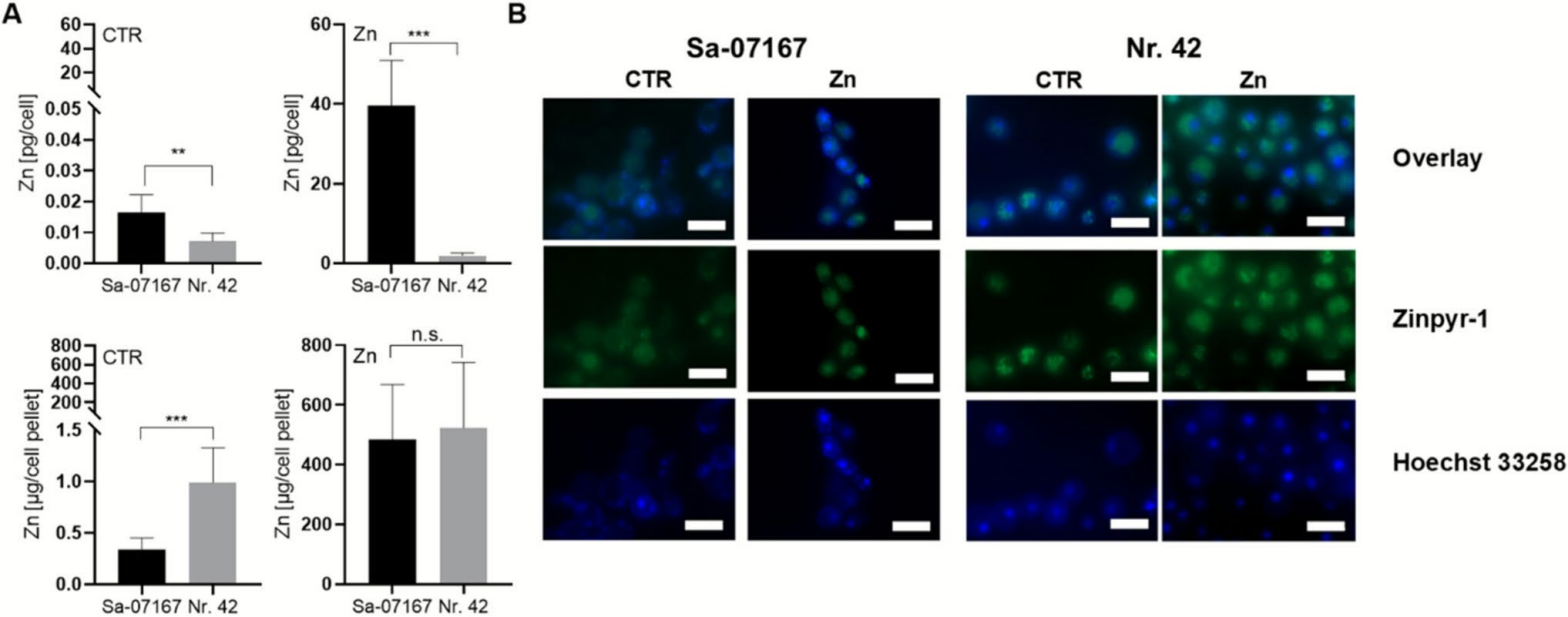
Zn-enrichment of *S. pastorianus* Nr. 42 and *S. cerevisiae* Sa-07167 under upscaled biofermenter conditions. CTR and ZnSO_4_-treated (Zn) yeast cells were taken at the end of the Biostat® bioreactor fermentations (see Fig. 3) (**A**) Total Zn contents of the CTR and ZnY were determined by ICP-MS and reported on a per-cell basis and for the cell pellet obtained from 1 mL of the overall 1 L Biostat® fermentation. Cell densities for these samples are illustrated in Fig. 3A. Data are shown as means ± SD of 3 replicates. Statistics: Mann-Whitney *U* or *t*-test (n.s., not significant; **p < 0.01; ***p < 0.001). (**B**) Representative fluorescence microscopy images illustrating subcellular patterns of labile Zn in CTR and ZnSO_4_-treated (Zn) yeast. Cells were co-stained with Zinpyr-1 (green), a low-molecular-weight Zn fluorescence sensor, and Hoechst 33258 (blue) for nuclear DNA. Overlay images illustrate the intracellular distribution of Zinpyr-1 fluorescence in relation to nuclear staining. Fluorescence was detected using the following filter settings: Zinpyr-1, λ_ex_ 450–490 nm/ λ_em_ 515–560 nm; Hoechst 33,258, λ_ex_ 365 nm/ λ_em_ 445/50 nm

ICP-MS measurement from the large-scale batch bioreactor samples showed Zn contents per cell nearly equivalent to microfermentations (Fig. 4A). The total Zn quantity present in the final *S. pastorianus* Nr. 42 biomass (523.7 ± 206.3 μg Zn/cell pellet) again exceeded that of *S. cerevisiae* Sa-07167 (484.8 ± 172.6 μg Zn/cell pellet) (Fig. 4A). As this Zn enrichment is based on different fermentation times and different cell numbers, the high Zn accumulation in *S. cerevisiae* Sa-07167 biomass after 1 h ZnSO_4_ incubation (total fermentation time 8.4 h) is due to the huge cellular Zn enrichment capacity of this strain (39.60 ± 8.96 pg Zn/cell; ~ 2500-fold increase compared to yeast grown in WMIX control medium). In contrast, in *S. pastorianus* Nr. 42, a 41.3 h fermentation in ZnSO_4_-upgraded WMIX media (total fermentation time 48 h) led to a rather moderate Zn content per cell (1.9 ± 0.6 pg Zn/cell; ~ 270-fold increase compared to control cells) but substantially higher total Zn-enriched biomass.

In addition to recording the total Zn, labile Zn (often also referred to as “rapidly exchangeable Zn” or “free Zn” in the literature) was measured, since this is considered a useful indicator of the Zn balance within yeast cells (Eide 2003; Devirgiliis et al. 2004; Nicola and Walker 2009; Pal et al. 2025; Ullah et al. 2025). The low- molecular weight Zn sensor Zinpyr-1 was used for live cell Zn imaging. Fluorescence images revealed substantial amounts of Zinpyr-1-accessible Zn in the cell interior for both strains with punctiform enrichments (Fig. 4B). Yeast fermented under excessive ZnSO_4_ treatment did not exhibit any changes in fluorescence intensity and distribution of Zinpyr-1. These observations suggest that alternative pathways for Zn buffering in both yeast strains may reduce the importance of vesicular compartments in Zn detoxification and storage.

## Zn-response proteome profiling

**Fig. 5:**
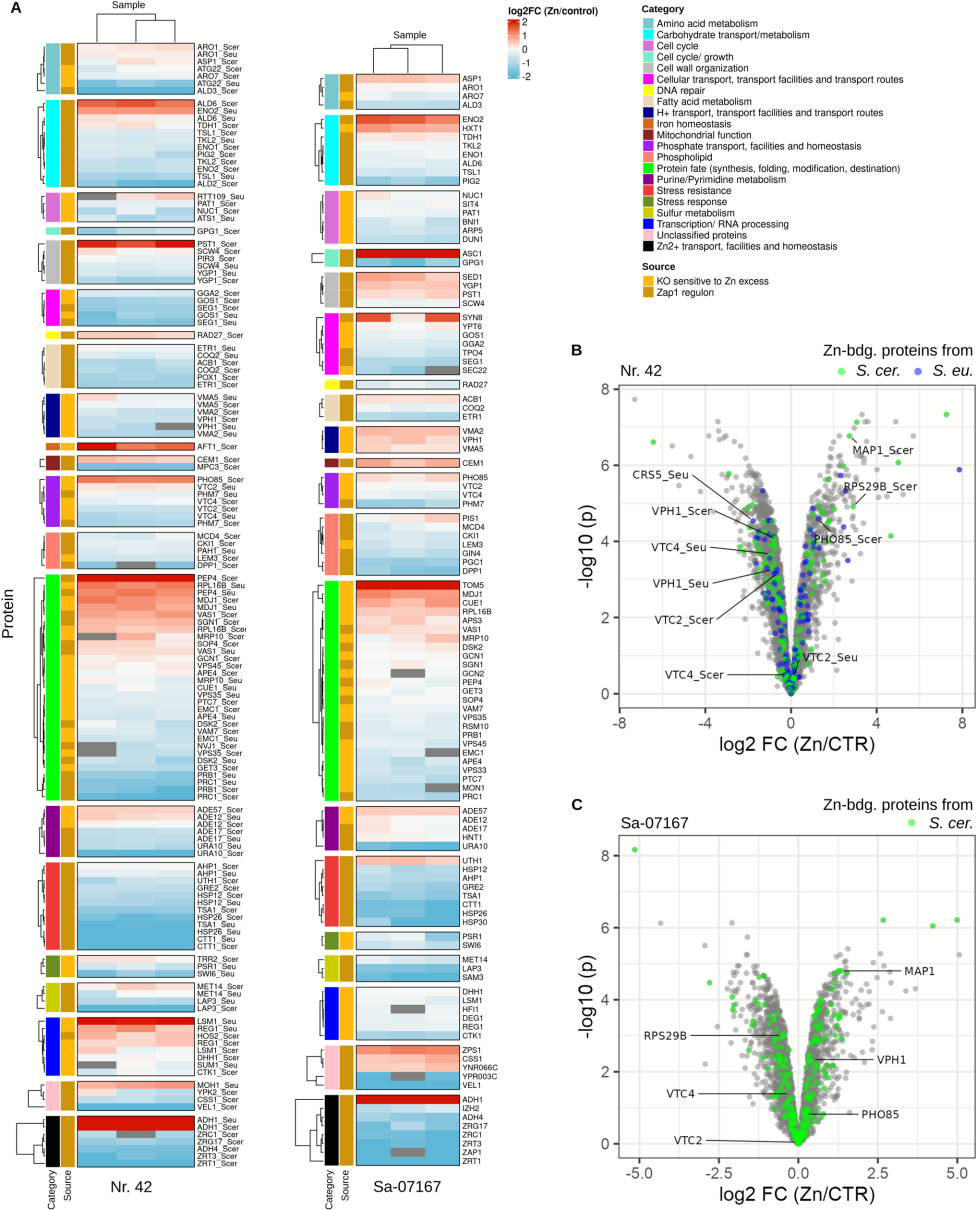
Changes in the proteome of *S. pastorianus* Nr. 42 and *S. cerevisiae* Sa-07167 as a result of ZnSO_4_ treatment. **A** Heatmap illustrating the expression of Zn-associated proteins in ZnY vs. CTR yeasts. Relative protein abundances (log2 FC Zn/CTR) are shown for three biological replicates of ZnY vs. the median abundance across three CTR replicates. Grey boxes indicate missing values. Proteins belonging to the ZAP1 regulon (as annotated by SGD, dark yellow) and those found to contribute to Zn tolerance in Zhao et al.’s knockout screen (light yellow) (Zhao et al. 2020) were included in the analysis. (**B** + **C**) Volcano plots highlighting differential expressions between Zn-treated and CTR samples. Log2 fold-changes and *p*-values (adjusted for multiple hypothesis testing) were determined using limma (Ritchie et al. 2015). Predicted zinc-binding proteins in the *S. cerevisiae* proteome (Wang et al. 2018) are highlighted

To shed light on Zn homeostasis in the two yeast strains, we investigated alterations in proteomes when fermented in either CTR or 10 mmol/L ZnSO_4_-enhanced WMIX media. Considering the highest Zn enrichments noted earlier, short-term incubation was selected for *S. cerevisiae* Sa-07167, while long-term fermentation was chosen for *S. pastorianus* Nr. 42. Both strains were cultivated in biological triplicates for each condition. Proteins were measured using a data-independent acquisition (DIA) method and processed using deep neural networks as implemented in DIA-NN (see material and methods). After quality filtering, we retained for *S. cerevisiae* Sa-07167 16,405 unique precursors that quantified 2871 proteins in both CTR and ZnY samples. We further detected 78 proteins solely in CTR samples, and 302 proteins solely in the ZnY for *S. cerevisiae* Sa-07167 samples (Supplementary Information Table S2a and 2b). For *S. pastorianus* Nr. 42, which is an interspecies hybrid of *S. cerevisiae* and *S. eubayanus*, we used a two-species processing approach to assign proteins detected in these samples unambiguously to either parent species. After quality filtering, we retained 10,572 unique precursors that quantified 3540 proteins present in CTR and ZnY *S. pastorianus* Nr. 42 samples, of which 1985 proteins mapped to *S. cerevisiae* and 1555 to *S. eubayanus*. 1050 proteins were present as orthologs, i.e., mapped to both *S. cerevisiae* and *S. eubayanus*, but showed sufficient sequence divergence to be identified as distinct proteins based on proteotypic peptides (Supplementary Information Table S3a). The high number of quantified orthologs explains the apparent difference in protein identifications between *S. cerevisiae* Sa-07167 and *S. pastorianus* Nr. 42 (2871 vs. 3540, respectively). For *S. pastorianus* Nr. 42, 170 proteins were detected solely in the CTR condition (84 mapping to the *S. cerevisiae* parent, 86 to the *S. eubayanus* parent), and 1274 proteins were detected solely in the Zn condition (674 mapping to the *S. cerevisiae* parent, 600 to the *S. eubayanus* parent). Of the 170 proteins detected only without Zn treatment, 8 were identified as *S. cerevisiae* and *S. eubayanus* orthologs, and of the 1274 proteins detected only under Zn stimulation, 153 such orthologs were identified (Supplementary Information Table S3b). RNA processing and metabolic biosynthesis of amino acids, amides, and organic acids were among the key functional categories enriched in response to ZnSO_4_ treatment (Supplementary Information Table S2c and S3c). The glycolysis and gluconeogenesis pathway also emerged as enriched, even though ZnSO₄ addition did not result in observable growth improvement (Fig. 3). Next, we evaluated the datasets for proteome alterations known to contribute to Zn homeostasis regulation in laboratory yeast strains. We searched for Zn-dependent gene products transcriptionally induced by Zap1 (“Zap1 regulon”, Fig. 5A), a key player in homeostasis of Zn-restricted yeast (Eide 2020). We further explored zinc tolerance determinants reported in a recent *S. cerevisiae* knockout screen (Zhao et al. 2020), proposing that they might support *S. cerevisiae* Sa-07167 and *S. pastorianus* Nr. 42 in handling Zn surplus (“KO sensitive to Zn excess”, Fig. 5A). Differences in protein expression between CTR and ZnY samples were visualized for both yeast strains separately. A clear pattern emerged with higher abundance of Zap1-regulated proteins in CTRs and an enhanced Zn excess signature in ZnYs (Fig. 5A and Supplementary Information Fig. S2). Zap1 was among the proteins detected solely in the *S. pastorianus* Nr. 42 CTRs (Supplementary Information Table S3b) and substantially higher in the *S. cerevisiae* Sa-07167 CTR samples (log_2_FC Zn/CTR: −2.8; Supplementary Information Table S2d). Other Zap1-transcriptionally regulated targets were also enriched in the CTR samples. The plasma membrane-localized, high-affinity Zn transporter Zrt1 was expressed outstandingly high under CTR fermentation conditions (log_2_FC Zn/CTR: −5.1 for *S. cerevisiae* Sa-07167; −6.4 for *S. pastorianus* Nr. 42 *S. cerevisiae* ortholog). Of the two low-affinity plasma membrane Zn uptake proteins, only Fet4 was detected in the *S. cerevisiae* Sa-07167 ZnY datasets. Zrt3 (Zn transporter responsible for mobilizing Zn stored in the yeast vacuole) and Zrc1 (transports Zn from cytosol to vacuole/secretory pathway) were more abundant in the CTR samples (log_2_FC Zn/CTR: −2.1 for Zrt3 and −2.0 for Zrc1 *S. cerevisiae* Sa-07167; −2.2 for Zrt3 and −0.9 for Zrc1 *S. pastorianus* Nr. 42 *S. cerevisiae* ortholog). The two Zn exporters serving the endoplasmic reticulum and secretory pathway were observed in *S. cerevisiae* Sa-07167, with Msc2 detected in Zn conditions only (Supplementary Information Table S2b). Zrg17 instead was higher in the CTR samples (log_2_FC Zn/CTR: −1.5 for *S. cerevisiae* Sa-07167). Overall, almost a third of the 144 gene products assigned in the *Saccharomyces* Genome Database (SGD) as Zap1 targets were higher in the CTR yeast cell proteomes (Supplementary Information Fig. S2). So Zap1 seemed to be activated in both strains for adaptation to the CTR medium to thrive under Zn-restricted growth conditions (0.8 µmol/L Zn supply by WMIX basal medium). Zn excess fermentation caused alcohol dehydrogenase (Adh) isozyme switching from Fe-dependent (Adh4) to Zn-dependent Adh isovariants (Adh1, Adh2, Adh3, and Adh5) (Fig. 5A). Expression levels of various other proposed Zn-binding proteins (Wang et al. 2018) were also increased in expression in response to ZnSO_4_ treatment (Fig. 5B and C). Ribosomal proteins explicitly highlighted as Zn-responsive by (Wang et al. 2018) and quantified in our dataset were clearly upregulated in the strain *S. cerevisiae* Sa-07167 and mostly upregulated or unchanged in strain *S. pastorianus* Nr.42 (Supplementary Information Fig. S3A and B). Taf1 was the only exception, being downregulated in Zn-treated samples in *S. cerevisiae* Sa-07167 and for the *S. cerevisiae* ortholog in *S. pastorianus* Nr.42, while the *S. eubayanus* ortholog showed minimal change. In tendency, proteins annotated with ribosome biogenesis, cytoplasmic translation, and ribosome, previously reported to decrease under Zn depletion (Wang et al. 2018), increased under Zn excess in both strains, with *S. cerevisiae* ortholog–mapped ribosome biogenesis proteins in *S. pastorianus* Nr.42 being the only group showing no change (Supplementary Information Fig. S3C). The metallothionein-like protein Crs5, which is discussed as a Zn-thionein for interim Zn storage in *S. cerevisiae* laboratory strains (Pagani et al. 2007; Nguyen et al. 2020), could be identified in *S. pastorianus* Nr. 42 samples but with decreased abundance in the ZnSO_4_ exposed cells (Supplementary Information Table S3d). Cup1 was not quantified in either strain because no unique proteotypic peptides could be assigned due to the presence of duplicated Cup1 paralogs. Nonetheless, many other proteins implicated in Zn binding and Zn stress response mechanisms identified in Zhao et al. (2020) genome-wide screens were also found to be increased in this study’s ZnYs. These proteins relate to metabolism, protein fate (synthesis, folding, modification, determination), H^+^ cellular transport, and phosphate homeostasis (Fig. 5A). There might be a connection between increased expression of the vacuolar H^+^-ATPase subunits Vma2, Vma5, and Vph1 in both yeast strains and the establishment of the proton gradient needed for Zn transport into the vacuoles. Inside the vacuoles, polyphosphates play a crucial role in Zn storage, though their presence has also been identified at the yeast cell's periphery (Simm et al. 2007; Nguyen et al. 2019; Kalebina et al. 2024).

In this study’s yeast strains, however, excess Zn led to the decrease of polyphosphate polymerase Vtc 2/4 protein expression. Pho85, a cyclin-dependent kinase involved in the yeast response to phosphate starvation (Choi et al. 2017; Ogawa et al. 2000), was higher in the ZnY proteomes. As a multifunctional kinase, Pho85 operates in several ways for the regulation of cellular reactions to nutrition; amongst others, this includes proper expression of several metal sensor genes and their regulatory gene (Nishizawa et al. 2004; Mao et al. 2003).

## Spectroscopic characterization of Zn-enriched freeze-dried yeast biomass

**Fig. 6:**
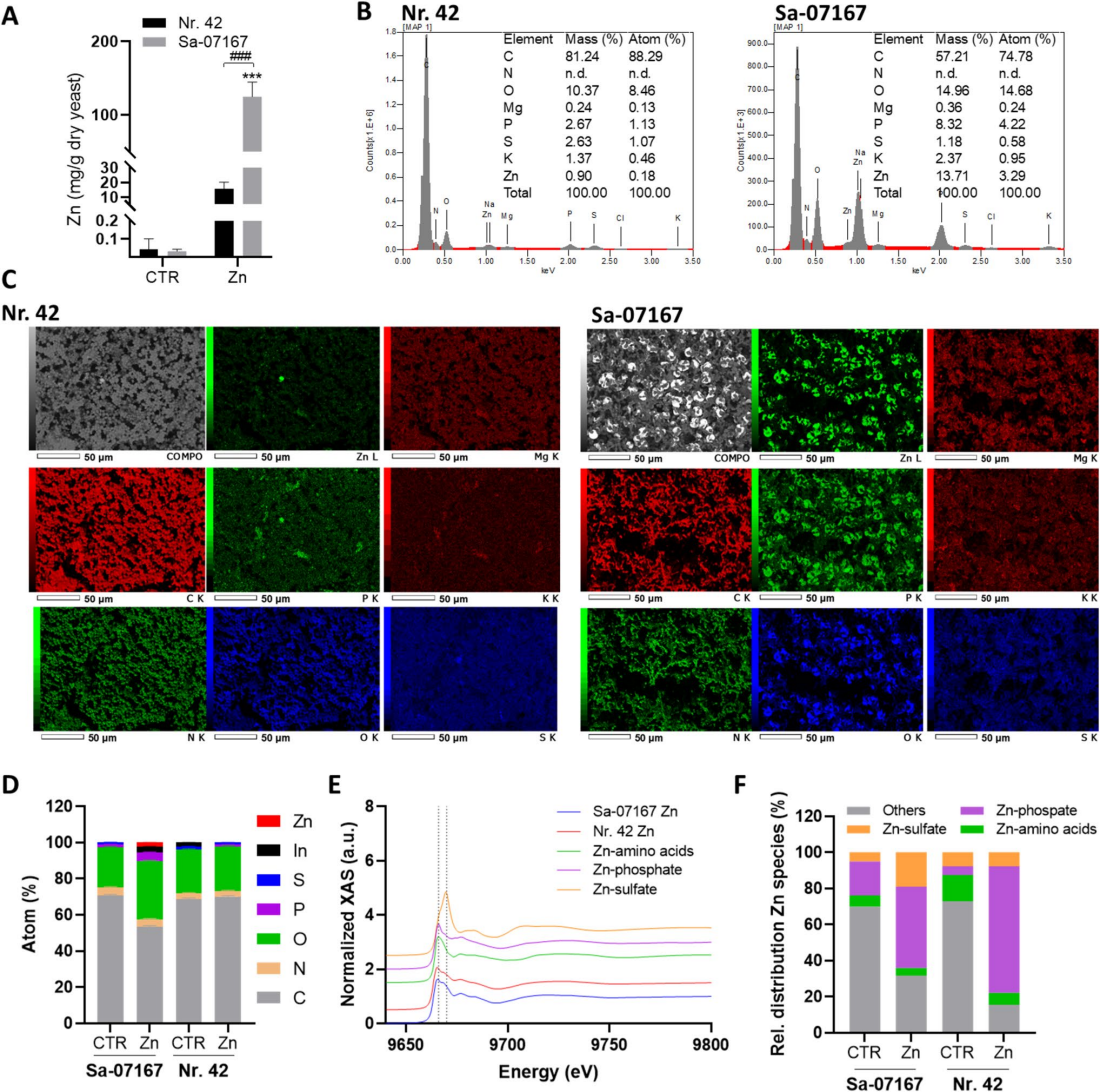
Metal analysis of the Zn-enriched freeze-dried yeast biomass. CTR and ZnY from fermentation runs in Biostat® bioreactors were freeze-dried and analyzed for (**A**) Total Zn content using ICP-MS. Two-Way ANOVA with Sidak’s multiple comparisons test was applied to test for statistical differences between one strain w/o (CTR) and after Zn-enrichment (***p < 0.001) and between both yeast strains (### *p* < 0.001). (**B**) SEM-energy-dispersive X-ray spectra including mass and atom percentage of selected elements. (**C**) SEM micrographs and EDX maps of the spatial distributions of Zn, Mg, C, P, K, N, O, S. Zn was detected using the L line of x-ray emission, while the other elements were detected using the K line. **D**) X-ray photoelectron spectroscopy elemental profiling. The traces of indium detected in the *S. pastorianus* Nr. 42 CTR samples originate from the indium adhesive foil used to tape the yeast powders. **E**) Zn K-edge XAS spectra for ZnY samples and Zn references. **F**) Results of the linear combination fit showing the relative distribution of Zn-species in the ZnY samples

The biomass harvested from Biostat® fermentation was freeze-dried to maintain the structural integrity of the cells (Supplementary Information Fig. S4). The ICP-MS measurements revealed ~ 0.03 mg Zn/g_dry yeast_ for the CTR and ~ 125 mg Zn/g_dry yeast_ for the Zn-enriched *S. cerevisiae* Sa-07167, while *S. pastorianus* Nr. 42 CTR contained ~ 0.04 mg Zn/g_dry yeast_ and ~ 15.7 mg Zn/g_dry yeast_ after Zn-enrichment (Fig. 6A). SEM–EDX confirmed these observations, detecting an average of 1.04 ± 0.16 wt.% Zn for *S. pastorianus* Nr. 42 and 10.35 ± 4.28 wt.% Zn for *S. cerevisiae* Sa-07167 in the freeze-dried ZnY powders (data from Fig. 6B and Supplementary Information Fig. S5A and B were used to calculate the average values). Interestingly, ZnY powders were also higher in phosphorus compared to CTR samples (Fig. 6B and Supplementary Information Fig. S5A and B). We further used ultra-high vacuum (UHV) XPS, a surface-sensitive quantitative spectroscopic technique based on the photoelectric effect for elemental characterization of cell surfaces (depth analyzed between 1–10 nm usually; (Baer et al. 2019)). The most common elements in microbial cell surfaces measured by XPS are carbon, nitrogen, oxygen, and phosphorus (Wei et al. 2021). These elements were also detected in most of the samples in the XPS measurements (except for phosphorus for *S. pastorianus* Nr. 42 CTR) (Fig. 6D). XPS spectra confirmed the presence of Zn in freeze-dried *S. cerevisiae* Sa-07167 ZnY samples (2.28 (at-) %). So, along with the microscopic observations from the microfermentations, we concluded that Zn occupies a considerable part of the surface of this yeast. Despite the high Zn amounts in *S. pastorianus* Nr. 42 ZnY samples measured by ICP-MS, no Zn peaks were detected by XPS, suggesting Zn’s storage more distantly within the cell interior.

Two-dimensional SEM–EDX image of Zn-enriched *S. cerevisiae* Sa-07167 depicts Zn- and P-rich precipitates localized on the cell surface (Fig. 6C). Considering this colocalization, it is quite likely that Zn is complexed with nearby phosphorus-containing ligands. This assumption is strengthened by the results of the XAS analysis (Fig. 6E). The spectra of ZnY in both strains show differences compared to ZnSO_4_ (Fig. 6E) and the linear combination fit resulted in a contribution of ZnSO_4_ lower than 10% for both strains (Fig. 6F), indicating changes in the Zn chemical environment during fermentation and/or freeze-drying due to a transformation of the Zn species. As a result of the linear combination fit, it can be concluded that Zn in the enriched yeast is coordinated by close proximity to P-O-ligands (45% in *S. cerevisiae* Sa-07167 and 70% in *S. pastorianus* Nr. 42) and to amino-acid-ligands (36% in *S. cerevisiae* Sa-07167 and 22% in *S. pastorianus* Nr. 42) in both strains (Fig. 6E + F).

### Zn-bioaccessibility from in vitro digested yeast

**Fig. 7:**
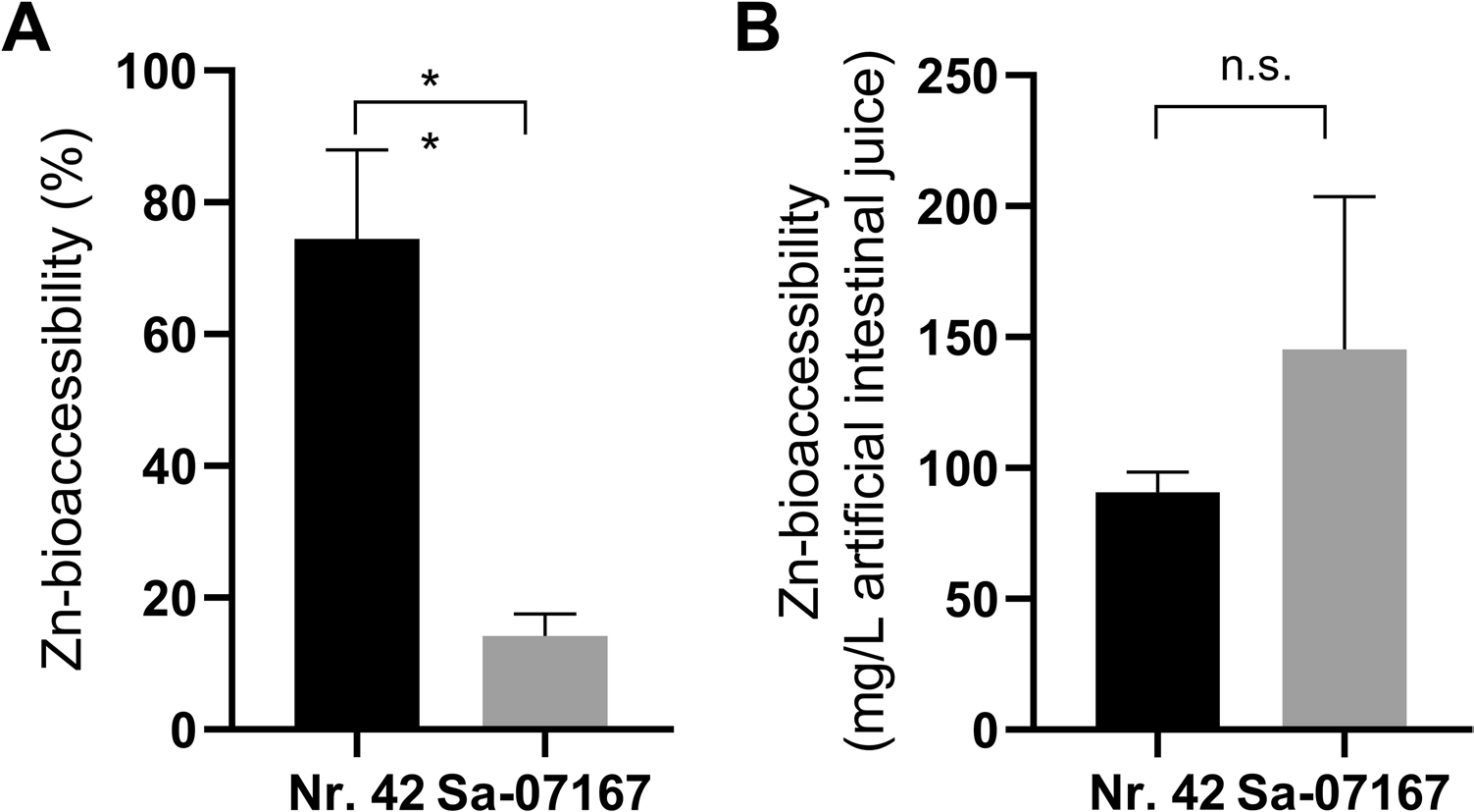
Zn-bioaccessibility from in vitro-digested freeze-dried ZnY. Percental bioaccessibility of Zn species (**A**) and Zn concentration (**B**) in the artificial intestinal juice supernatant after enzymatic digestion of ZnY. Shown are mean + SD from three replicates. Statistical differences between both strains were tested using an unpaired *t*-test (n.s., not significant; **p < 0.01)

The release of Zn from freeze-dried yeast powders was determined using an in vitro human gastrointestinal digestion model. The results show clear differences between both strains, with *S. cerevisiae* Sa-07167 Zn-bioaccessibility being significantly lower (14.23 ± 2.67%) than that of *S. pastorianus* Nr. 42 (74.43 ± 11.02%) (Fig. 7A). Considering the total Zn content of the ZnYs (see Fig. 6A), enzymatic digestion yielded higher Zn concentrations in the intestinal juice from *S. cerevisiae* Sa-07167 ZnY (145 mg Zn/L) compared to the digest of the freeze-dried ZnY powder from *S. pastorianus* Nr. 42 (90 mg Zn/L).

## Discussion

Zn as an essential micronutrient has pivotal roles in various processes of the human body, (Hambidge 2000). Accordingly, Zn deficiency can have major consequences including elevated childhood mortality, stunted growth, impaired immune function, and cognitive dysfunction (Lowe et al. 2024). With around 17% of the world population at risk for Zn deficiency, corresponding to approximately around 1.1 billion people (Hall and King 2022; Lowe et al. 2024; Kumssa et al. 2015), and the inability of the human body to store extra Zn, it is important to maintain a healthy Zn homeostasis in the world’s population (Haase and Rink 2014). Zn-enriched yeasts are attracting growing interest in the global food supplement market, though in Europe, regulatory approval still hinges on a conclusive risk assessment.

### Evaluating Zn-enrichment across the industrial yeast strains

From EFSA’s point of view, more comprehensive data is needed on the Zn species present in the yeast and the metabolic fate of the supplied Zn in order to evaluate the safety of the use and consumption of ZnY in foods, including food supplements for the general population (EFSA 2009a). The seemingly simple questions of Zn speciation and its availability pose a challenge for applicants seeking EFSA’s authorization of ZnY, given the notable variability in yeast responses to Zn treatments (Zhao et al. 2020; Aulakh et al. 2025). Individual levels of protein expression in domesticated yeast strains from various industries indicate a modulation that is affected by genetic characteristics as well as the technological surroundings (Davydenko et al. 2020). Thus, it is not easily predictable which mechanisms are dominant and hence formative for Zn speciation and enrichment outcomes. The screening of industrial yeasts in this study revealed that in the early exponential phase, the Zn per-cell content of Zn-exposed cells peaked after one hour of incubation, while with continuing fermentation, a decrease in the Zn content occurs. Several reasons might explain this observation, one being Zn accumulation near the surface with subsequent desorption. For some of the ZnYs, flocculation—a property especially observed for, but not limited to, some brewery yeasts (Nayyar et al. 2014; Stewart 2018)—was visible under the microscope, indicating some extent of interaction between Zn and cell surface molecules. Another reason might be that with ongoing fermentation, extracellularly localized Zn can increasingly interact with molecules excreted during fermentation, resulting in the formation of insoluble Zn species in the nutrient solution or yeast surface proximity (biomineralization) (Debnath et al. 2024; Li and Li 2022) and thus a decrease in measured yeast Zn contents. It is also conceivable that Zn is actively taken up in the early exponential phase and subsequently excreted via the secretory pathway as part of a homeostatic response (Bird and Wilson 2020). Future research involving pulse-chase experiments (Nies et al. 2024) will be essential for analyzing the processes of Zn uptake and release, as well as the turnover of Zn ions within the cell. Comparing the Zn concentrations in our ZnYs to the literature, *S. pastorianus* Nr. 42 accumulated Zn in a similar magnitude as yeasts from two other studies reporting 4 mg Zn/g dry yeast (Azad et al. 2014; Wang et al. 2011). Meanwhile, total enriched Zn/dry matter of *S. cerevisiae* Sa-07167 reached almost 7 times higher amounts than the 18.5 mg Zn/g dry yeast reported before (Šillerová et al. 2012). Zn enrichment of various *Saccharomyces* and non-*Saccharomyces* strains performed by (García-Béjar et al. 2020) resulted in Zn contents from 10 pg Zn/cell in the intracellular fraction, comparable to the Zn concentration of Sa-07167 in our study. Most recently, Zare et al. (2025) reported a remarkably high Zn accumulation capacity of (700 ± 60) mg Zn/dry biomass for *Candida* sp. pz46 strain S46. Besides these strains with strong Zn-accumulating capacities, Zn-enriched *S. cerevisiae* Sa-07167 showed a Zn level above most records for Zn-enriched yeasts so far.

### Proteomic shifts under Zn variation and speciation considerations

To cope with the elevated Zn concentrations, the cell must coordinate multiple mechanisms to prevent harmful effects. The proteomic data suggested that while CTR yeasts rely on Zap1-regulated expression, ZnY activates alternative protective pathways, reflecting Zn excess adaptation. The metal-responsive regulatory protein Zap1 has a central role in *S. cerevisiae* response to Zn deficiency (Eide 2009, 2020; Zhao and Eide 1997; Wu et al. 2008). Recent studies from the Eide group indicated that Zn deficiency impairs glycolysis, mainly through Zap1-controlled alterations in enzyme expression, contributing to reduced growth in *S. cerevisiae* laboratory BY4741 and BY4742 strains (MacDiarmid et al. 2024). The observed similarities in growth, glucose utilization, and ethanol production (data not shown) between CTR and Zn conditions indicate that these Zap1-dependent responses were not active in the industrial yeast strains fermented in WMIX basal medium. However, other Zap1-regulated proteins were higher in abundance in the CTR samples. In Zn-limited cells, Zrt3 is up-regulated; a gene encoding a protein responsible for transporting Zn from the vacuole into the cytosol, thereby making it available for utilization. At the same time, Zrc1 is upregulated by Zap1, encoding a protein responsible for Zn transport into the vacuole and therefore mediating Zn tolerance. In limited Zn conditions, this increased expression of Zrc1 is a pro-active mechanism for Zn storage (MacDiarmid et al. 2003), as the high levels of Zn transporter proteins in the plasma membrane can lead to a rapidly increasing cytosolic Zn concentration once Zn is available (Eide 2009, 2020). In the CTR samples of *S. cerevisiae* Sa-07167 and *S. pastorianus* Nr. 42, Zrt3 and Zrc1 were also higher abundant. All these observations indicate that the Zap1 regulon was activated in the CTR samples of both strains, enabling the cells to adapt to the Zn-restricted growth conditions of 0.8 µmol/L Zn supplied by WMIX basal medium. Under these Zn-limited conditions, Zap1 binds Zn-responsive elements (ZREs) in target promoters via its DNA-binding domain, while its AD1 and AD2 domains recruit coactivators, such as SWI/SNF and SAGA, to remodel chromatin and enhance transcription (Eide 2020). Zap1 in these industrial yeast strains may exhibit some degree of functional variation, potentially arising from genetic diversity that is known to occur in natural and domesticated yeast genomes (Peter et al. 2018; Loegler et al. 2024; Jakobson et al. 2025), which could also contribute to differences in the Zap1 response between *S. cerevisiae* Sa-07167 and *S. pastorianus* Nr. 42. At present, no research has directly explored the impact of *ZAP1* polymorphisms on metal homeostasis in industrial strains, but their possible occurrence points to an interesting direction for future investigation. Interestingly, although proteomic data suggest that yeast cells from both strains are experiencing Zn deficiency when grown in WMIX basal medium, Zn levels in CTR yeasts ranged from approximately 1–60 fg/cell (equivalent to ~ 1–55 × 10^7^ atoms of Zn per cell), which is at or above the estimated minimum requirement of ~ 1 × 10⁷ Zn atoms per cell for optimal yeast growth (Wang et al. 2018).

After supplementation of 10 mmol/L ZnSO_4_, surplus defensive mechanisms were activated in the ZnY cells. Proteomics showed that Zn excess fermentation caused Adh isozyme switching from Adh4 in the CTR samples to Adh1, Adh2, Adh3, and Adh5 isovariants in the ZnY samples, as already observed in *S. cerevisiae* and non-model yeast species (Eide 2009; Wang et al. 2018), pointing to an adaptation for internal Zn storage. Wang et al. (2018) concluded from their study that beside Adh1, just a few, but highly abundant proteins account for a big part of total Zn storage capacity in Zn-replete *S. cerevisiae*. More specifically, they deduced that almost 90% of the total Zn requirement in Zn-sufficient cells is represented by the twenty most abundant Zn proteins (Wang et al. 2018). This study’s proteomics also showed a relative increase in the number of potentially Zn-binding proteins in ZnY samples, and their Zn^2+^ metalation could aid in sequestering surplus Zn, thereby minimizing its harmful impact. Within this context, the roles of the metallothionein-like proteins Cup1 and Crs5 as Zn binders remain a topic of debate. Of those two, Cup1 has the leading role in Cu detoxification, while Crs5, having important constitutive expression levels, coordinates Cu(I) ions in a kinetically more labile and solvent-accessible way. This suggests alternative physiological roles of Crs5 in the metabolism of essential metals like Zn (Pagani et al. 2007). In the present study, Crs5 was identified in *S. pastorianus* Nr. 42 samples but with decreased abundance in the ZnSO_4_ exposed cells which suggests a minor role in Zn trafficking and Zn homeostasis for the strains analyzed under the present conditions. According to Wang the Zn proteome in replete cells translates to 9× 10^6^ Zn-binding sites on proteins per cell, which is almost equivalent to the minimal Zn quota required for optimal cell growth. They found the total Zn content in replete cells to be 2.3 × 10^7^ atoms per cell, while Simm reported a high amount of vacuolar accumulated Zn, which corresponded to ~ 7 × 10^8^ atoms of vacuolar Zn per cell. Even when both Zn pools were combined as an estimate of Zn-binding capacity, the resulting value still fell short of explaining the elevated Zn in this study’s ZnY samples (365 × 10^9^ atoms Zn/cell for *S. cerevisiae* Sa-07167 and 17 × 10^9^ atoms Zn/cell for *S. pastorianus* Nr. 42), indicating additional storage mechanisms. Our findings support existing evidence that yeast cells maintain a labile Zn pool (Eide 2003; Devirgiliis et al. 2004; Nicola and Walker 2009; Pal et al. 2025; Ullah et al. 2025), where Zn is loosely bound to low-molecular organic and inorganic ligands (Krężel and Maret 2016). This reservoir did not increase substantially under conditions of Zn excess. The XPS and SEM–EDX experiments on the freeze-dried yeasts showed substantial amounts of Zn localized close to the cell surface for *S. cerevisiae* Sa-07167. XAS analysis with a linear combination fit indicated that the Zn environment of the ZnY has changed from supplemented ZnSO_4_ to Zn-phosphate and Zn-amino acid species during fermentation and/or freeze-drying. Apart from XAS, SEM–EDX images also pointed towards the relevance of phosphate-containing molecules interacting with Zn. These could potentially be polyphosphates, albeit the abundance of the proteins belonging to the polyphosphate polymerase (Vtc) complex is rather decreasing in the ZnY samples. Polyphosphates can accumulate to high levels in yeast cell compartments, especially within the vacuole (Gerasimaitė and Mayer 2017; Yang et al. 2017), but were also described to localize on the cell periphery (Kalebina et al. 2024), and they are known to bind Zn with a high affinity (van Wazer and Campanella 1950). Deletion of genes related to phosphate transport and polyphosphate formation led to Zn sensitivity in *S. cerevisiae*, indicating that phosphate balance contributes to Zn homeostasis to handle Zn stress (Zhao et al. 2020). In liquid chromatography (LC)-ICP-MS data presented by Nguyen (Nguyen et al. 2019) a significant amount of vacuolar Zn appeared in a low-molecular-weight, phosphatase-sensitive fraction, likely representing Zn complexed with polyphosphates. A different study did not observe an influence of a disrupted phosphatase synthesis on vacuolar Zn storage and deduced that if polyphosphates are of importance, they only have a minor role as Zn-binding ligands (Simm et al. 2007). XPS analysis of *S. cerevisiae* Sa-07167 showed a certain fraction of Zn as well as phosphorus located near the cell surface. Beside the aforementioned polyphosphates, phosphomannans that emanate from the outer cell surface layer could contribute to the observed signal (Baek et al. 2024). Yet, another possibility is that these are biomineralized Zn-phosphate precipitates. It has been previously observed that yeasts can undergo biomineralization when phosphate and metals like Cu, Fe, or heavier elements such as U, Ce, and Yb accumulate locally on or within the cells (Ojima et al. 2020; Zheng et al. 2022; He et al. 2009).

Although the SEM images give no indication of cell wall damage, it cannot be ruled out that a restructuring of the cell walls has occurred as a result of the freeze-drying of the cells (Köhler et al. 2020), which could be accompanied by a leakage of phosphate-containing molecules. However, it can be assumed that numerous chemically and structurally distinct phosphate sites participate in Zn coordination (Chen and Wang 2008) inside cells and on their outer surfaces. In order to get a closer look at cell surface parameters without the risk of a collapsed cell structure due to sample preparation like freeze-drying, a NAP-XPS (near ambient pressure X-ray photoelectron spectroscopy) analysis of fresh cell samples could show which ligands are available for Zn biosorption and biomineralization close to the cell surface (Kjærvik et al. 2021). Further experiments are needed to clarify the cellular and subcellular distribution of Zn, for which nanoscale secondary ion mass spectrometry (NanoSIMS) or X-ray fluorescence (XRF) microscopy being methods allowing analysis at nanometer resolution. Complemented with analytical methods like transmission electron microscopy (TEM) or transmission X-ray computed tomography (XCT) providing images of the overall cellular and subcellular structures, NanoSIMS and XRF can display Zn localization as well as colocalization of other elements on elemental maps of single yeast cells (Penen et al. 2016; Lin et al. 2024; Chevrier et al. 2025; Ullah et al. 2025). The integration of synchrotron radiation X-ray absorption near edge structure (XANES) mapping could enable a more complete depiction of Zn speciation throughout cellular regions, offering deeper insights into its chemistry within different industrial strains under varied fermentation conditions (Ortega 2011; Thomas et al. 2019).

### Yeast-based Zn: Nutritional Perspective

Considering the nutritional application of Zn-enriched yeasts for the general population, around 80 mg of the freeze-dried Sa-07167 ZnY and 730 mg of the Nr. 42 ZnY powder would be more than sufficient to meet current reference values for daily dietary Zn intake of 7–10 mg (Haase et al. 2020). For Zn to be bioavailable, it must first be released from the yeast matrix, which is determined by the bioaccessibility (Maares et al. 2022; Tokarczyk and Koch 2025). According to our experiments, Zn bioaccessibility from the two ZnYs Sa-07167 and Nr. 42 is in a similar range as other common Zn supplements (Maares et al. 2022). Given that especially *S. cerevisiae* Sa-07167 has accumulated a significant fraction of Zn close to the cell surface with simultaneous high amounts of phosphorus, the aforementioned possible mineralization of Zn phosphate might be a reason for a poorer bioavailability (Seal and Heaton 1983). To ensure accurate assessment of zinc absorption from ZnY, large-scale screening must incorporate in vitro digestion–absorption models that mimic human gastrointestinal conditions (Maares et al. 2022), followed by human testing (Pellowski et al. 2024; Jäger et al. 2024).

## Conclusion

This study demonstrates that there are significant differences between industrial yeast strains in response to Zn excess regarding their fermentation behavior and Zn accumulation capacity. Proteomic profiling revealed a shift from an induced Zap1-regulon in CTR cells to a Zn excess response in ZnY, which indicates that in Zn excess conditions, some of the accumulated zinc is internalized into the cells. All ten industrial strains tested demonstrated significantly higher Zn accumulation under both microfermentation and biofermenter conditions compared to laboratory-scale cultivation experiments reported in the literature. Future studies should therefore place greater emphasis on fermentation process control and media selection when aiming for trace element enrichment. Speciation analysis with XAS showed P-O-ligands as well as amino acids as important Zn-coordinating molecules in both strains. The relationship between Zn and phosphate homeostasis in ZnYs needs to be further investigated to gain more insight into the cellular Zn storage pools with respect to the distribution and species of accumulated Zn. For this, advanced imaging methods like NanoSIMS or XRF microscopy along with sophisticated NanoXANES speciation analysis could be of great value to visualize element distributions and fingerprint Zn speciation at subcellular resolution – thereby contributing to a better understanding of the fate of accumulated Zn in selected industrial yeast strains. Nevertheless, EFSAs demand for more comprehensive data regarding Zn homeostasis, including characterization of Zn distribution pathways, specification of Zn species, as well as elucidation of the metabolic fate of Zn released from the ZnY in the human body, might remain challenging to meet. This is particularly true given that, depending on the genetic characteristics and the technological background of the yeast strain, it is generally difficult to predict the dominating homeostatic mechanisms and consequently the formed Zn species.

## Supplementary information

Below is the link to the electronic supplementary material.

ESM 1 (DOCX 2.79 MB)

ESM 2 (XLSX 953 KB)

## Data Availability

The mass spectrometry proteomics data have been deposited to the ProteomeXchange Consortium via the PRIDE partner repository with the dataset identifier PXD065844. Other data presented in this study are available on request from the corresponding authors.
