# Supplementary material for: Unlocking the Zn-enriching potential of industrial yeast strains—an experimental journey from metal analysis to proteomics
Source: Appl Microbiol Biotechnol. 2026 Jan 10;110(1):12. doi: 10.1007/s00253-025-13692-y (PMC12791086; doi:10.1007/s00253-025-13692-y)
Supplement: Supplementary file 1 — (DOCX 2.79 MB) [file 253_2025_13692_MOESM1_ESM.docx]

**Applied Microbiology and Biotechnology**

**Supplementary Material**

**Unlocking the Zn-enriching potential of industrial yeast strains – an experimental journey from metal analysis to proteomics**

Gina Grimmer ^1,2,3,#^, Julia Muenzner ^4,#^, Maximillian Schmacht ^3^, Maria Angels Subirana ^5^, Iris H. Valido ^5,6^, Philip Nickl ^7^, Paul M. Dietrich ^8^, Ievgen S. Donskyi ^7^, Dirk Schaumlöffel ^5^, Martin Hageböck ^3^, Michael Mülleder ^9^, Markus Ralser ^4,10,11,12^, Hajo Haase ^2^, Martin Senz ^3^, Maria Maares ^1,2,^*, Claudia Keil ^2,^*

1. Department of Food Chemistry, Institute of Nutritional Science, University of Potsdam, Arthur-Scheunert-Allee 114–116, 14558 Nuthetal, Germany
2. Department of Food Chemistry and Toxicology, Institute of Food Technology and Food Chemistry, Technische Universität Berlin, Straße des 17. Juni 135, 10623 Berlin, Germany
3. Versuchs- und Lehranstalt für Brauerei in Berlin (VLB) e.V., Department Bioprocess Engineering and Applied Microbiology, Seestraße 13, 13353, Berlin, Germany
4. Charité Universitätsmedizin Berlin, Department of Biochemistry, 10117 Berlin, Germany
5. CNRS, Université de Pau et des Pays de l’Adour, Institut des Sciences Analytiques et de Physico-Chimie pour l’Environnement et les Matériaux (IPREM) UMR 5254, Hélioparc, 2 avenue Pierre Angot, 64053 Pau, France
6. GTS Research Group, Department of Chemistry, Faculty of Science, Universitat Autònoma de Barcelona, Cerdanyola del Vallès, 08193 Barcelona
7. Institut für Chemie und Biochemie, Freie Universität Berlin, Takustr. 3, 14195 Berlin, Germany
8. SPECS Surface Nano Analysis GmbH, Voltastrasse 5, 13355 Berlin, Germany
9. Charité Universitätsmedizin Berlin, Core Facility – High Throughput Mass Spectrometry, Berlin, Germany
10. Centre for Human Genetics, Nuffield Department of Medicine, University of Oxford, Oxford, UK
11. Berlin Institute of Health at Charité - Universitätsmedizin Berlin, Berlin, Germany
12. Max Planck Institute for Molecular Genetics, Ihnestrasse 73, 14195 Berlin, Germany

***** Correspondence: Claudia Keil (c.keil@tu-berlin.de, Tel.: +49 (0) 30 31472701); Maria Maares (maria.maares.1@uni-potsdam.de, Tel.: +49 (0) 33200 88-5580)

- These authors contributed equally.

**
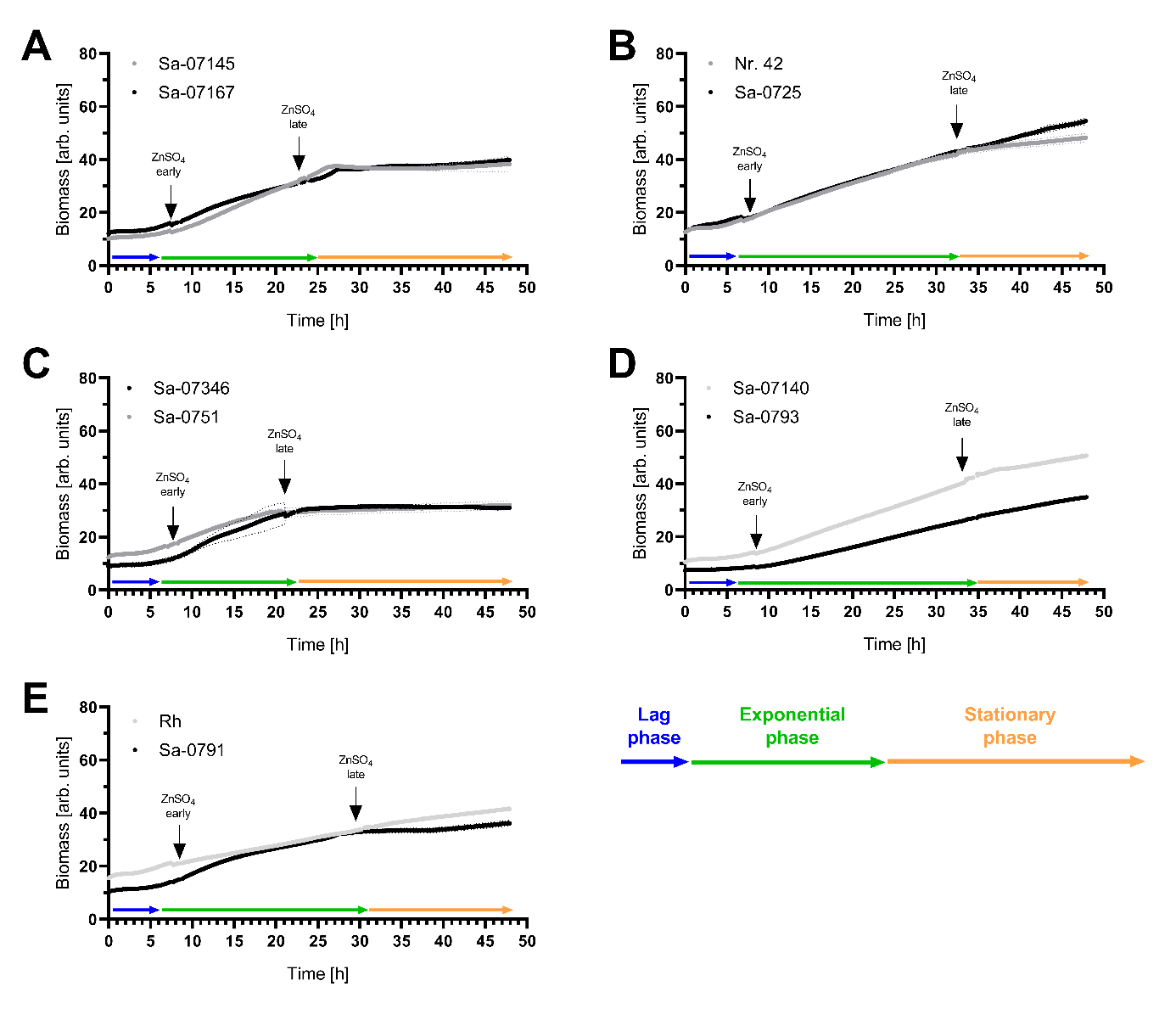
**

**Suppl. Figure S1: Growth curves of all ten yeast strains in WMIX medium in microtiter format of the BioLector®Pro.** Monitored yeast growth over the incubation period measured online by scattered light intensity. Based on these experiments, the time of ZnSO_4_ addition in the early or late exponential phase for subsequent experiments (marked with an arrow) was determined.


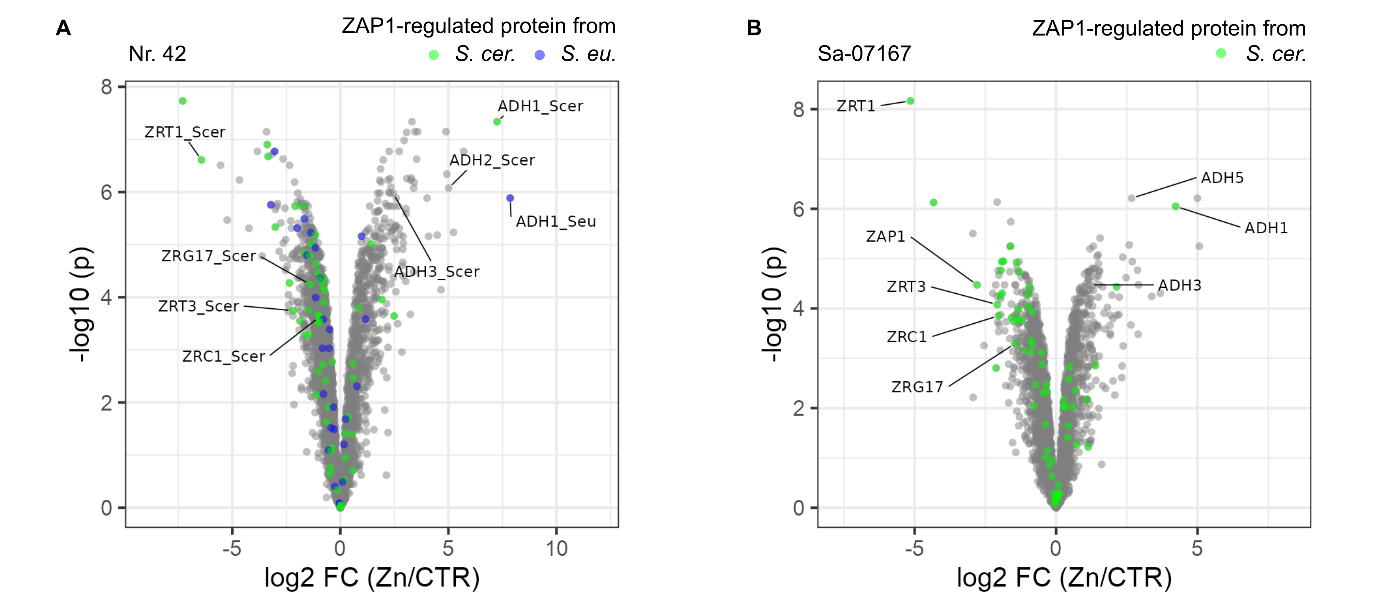


**Suppl. Figure S2: Differential protein expression for *S. pastorianus* Nr. 42 (A) and *S. cerevisiae* Sa-07167 (B) for ZnSO_4_ vs. CTR treatment highlighting ZAP1-regulated proteins.** Log2 fold-changes and *p*-values (adjusted for multiple hypothesis testing) were determined using limma version 3.63.2 (Ritchie et al. 2015). Proteins belonging to the ZAP-1 regulon are highlighted in the volcano plots.

**
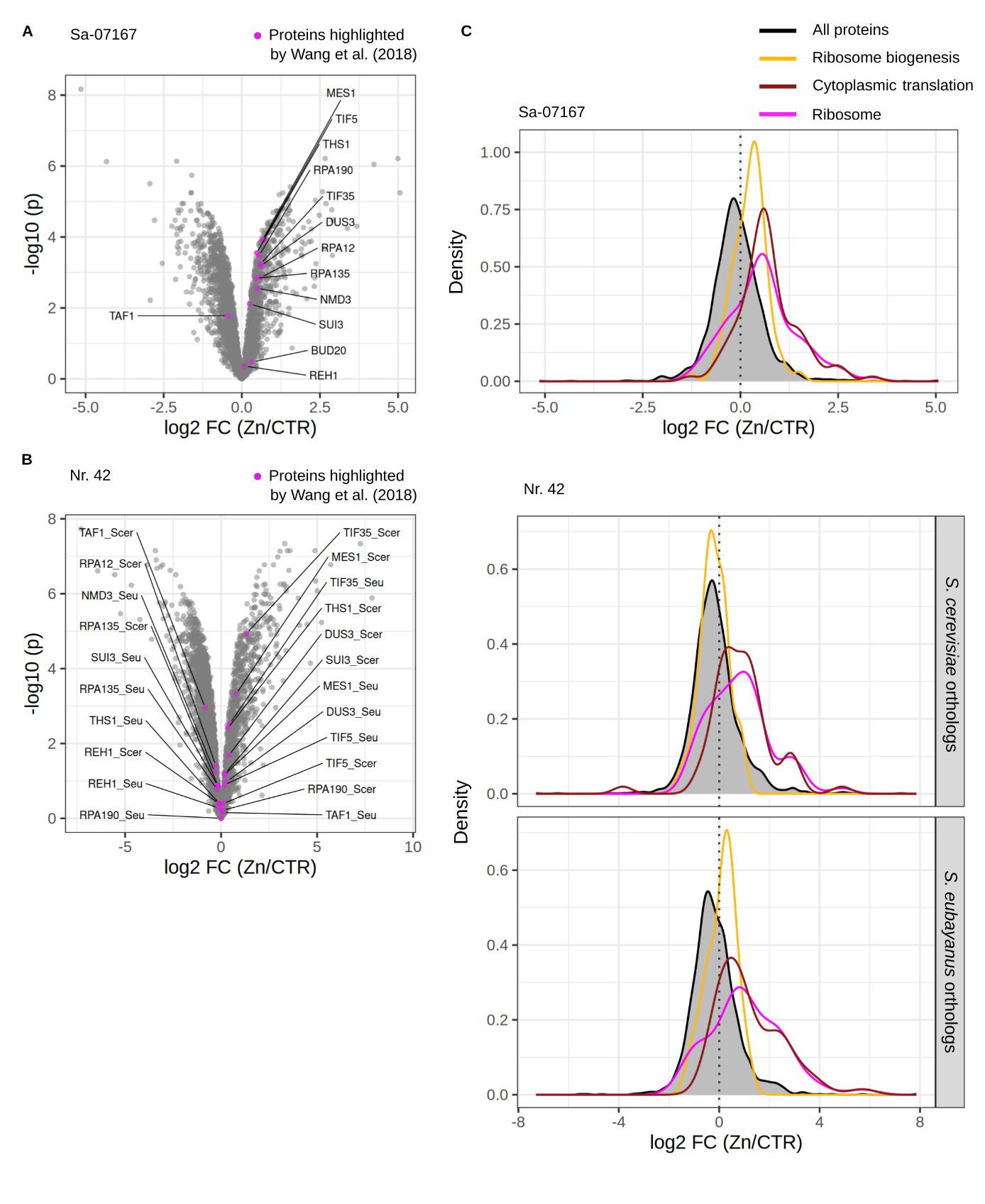
Suppl. Figure S3: Differential protein expression for *S. pastorianus* Nr. 42 and *S. cerevisiae* Sa-07167 for ZnSO_4_ vs. CTR treatment with a focus on ribosome-related processes.** Log2 fold-changes and *p*-values (adjusted for multiple hypothesis testing) were determined using limma version 3.63.2 (Ritchie et al. 2015).. (A/B) Proteins involved in ribosome-related processes and specifically identified as Zn-responsive in *S. cerevisiae* by Wang et al. (2018) are highlighted in the volcano plots. (C) Distributions are shown for all proteins (black), and proteins annotated to the KEGG terms "ribosome biogenesis" (orange), "Ribosome" (pink), and the GO term "cytoplasmic translation" (red). For strain *S. pastorianus* Nr. 42, distributions are shown separately for *S. cerevisiae* and *S. eubayanus*-mapped orthologs.

**
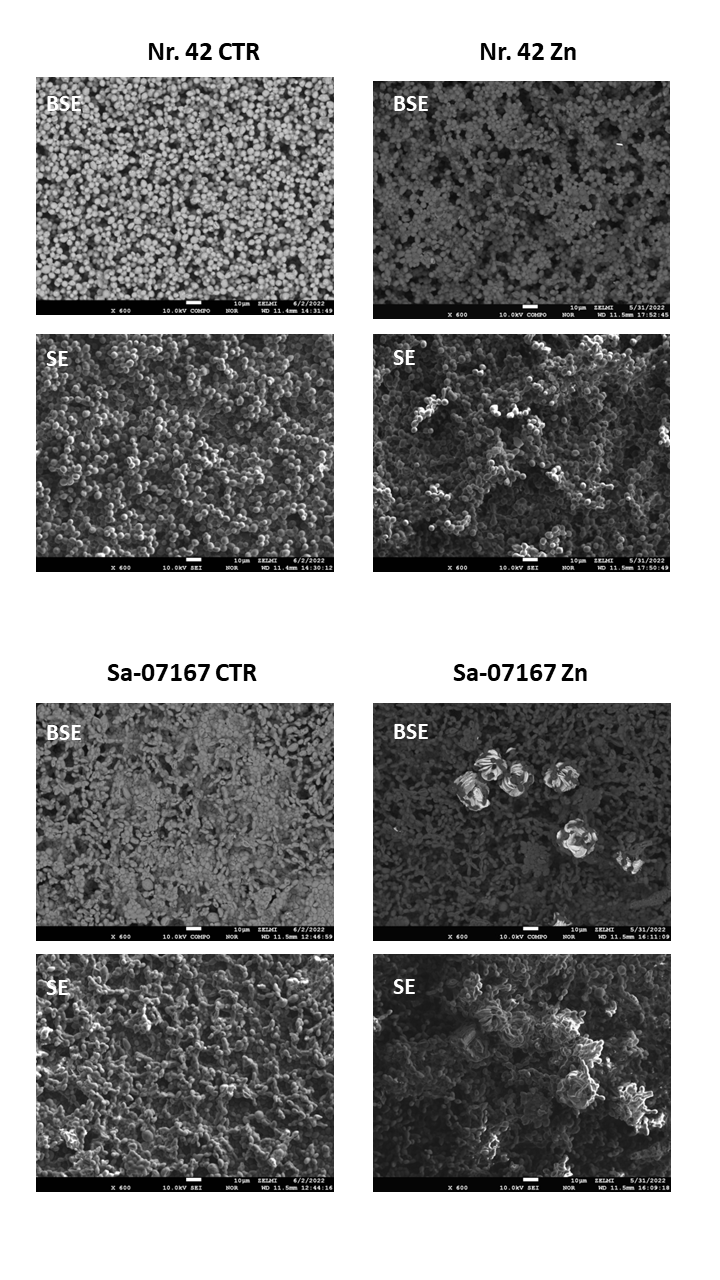
**

**Suppl. Figure S4: Backscattered electron (BSE) and secondary electron (SE) scanning electron microscope (SEM) images of freeze-dried CtrYeast and ZnYeast (600x magnification).** Scale bar = 10 μm.


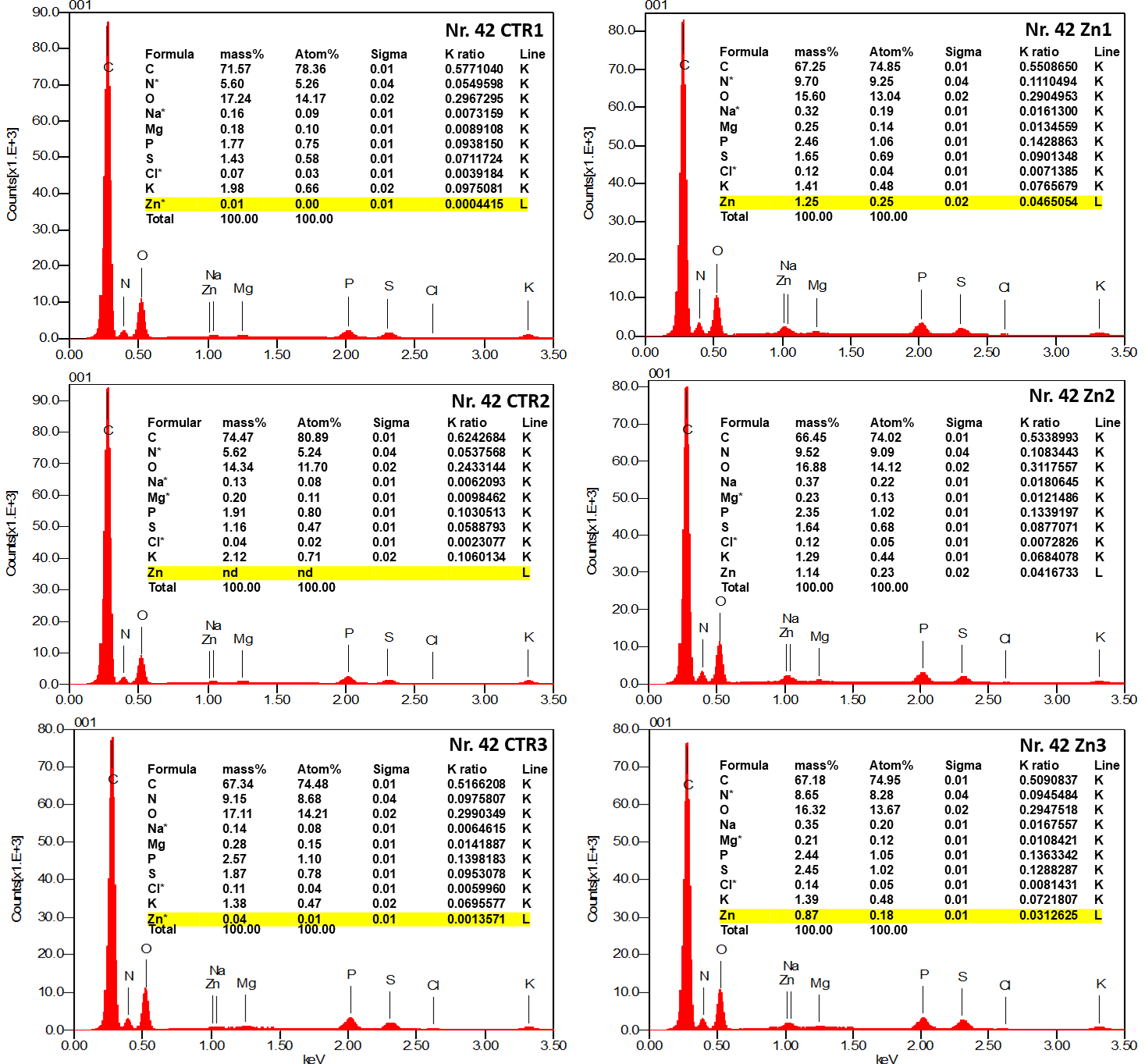


**Suppl. Figure S5A. Replica EDX spectra of freeze-dried *S. pastorianus* Nr. 42 CTR and ZnY samples.**

**
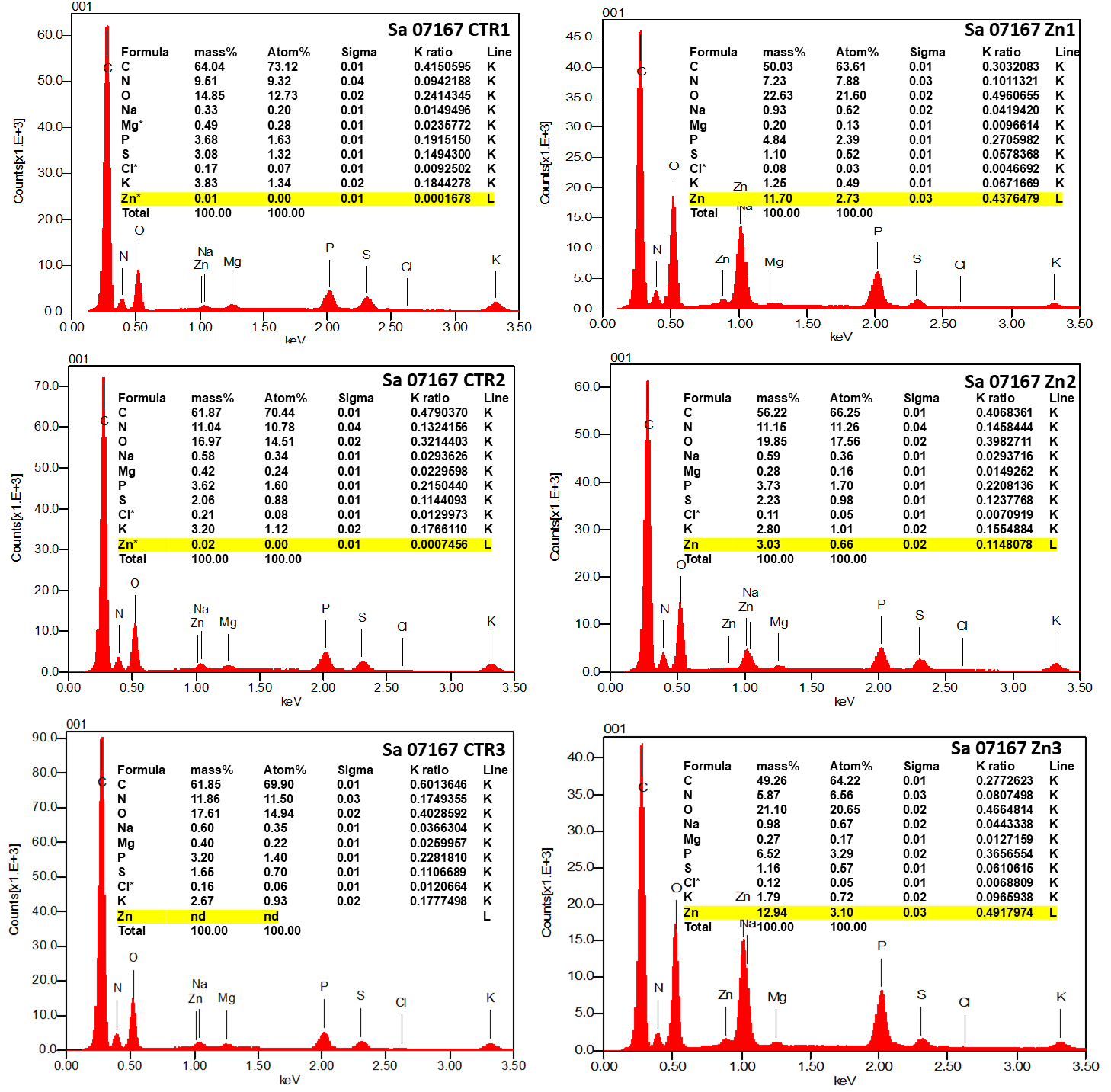
**

**Suppl. Figure S5B. Replica EDX spectra of freeze-dried *S. cerevisae* Sa-07167 CTR and ZnY samples.**

**References**

Ritchie ME, Phipson B, Di Wu, Hu Y, Law CW, Shi W, Smyth GK (2015) limma powers differential expression analyses for RNA-sequencing and microarray studies. Nucleic Acids Res 43/7:e47. doi: 10.1093/nar/gkv007
